# The Microbial Community of a Passive Biochemical Reactor Treating Arsenic, Zinc, and Sulfate-Rich Seepage

**DOI:** 10.3389/fbioe.2015.00027

**Published:** 2015-03-06

**Authors:** Susan Anne Baldwin, Maryam Khoshnoodi, Maryam Rezadehbashi, Marcus Taupp, Steven Hallam, Al Mattes, Hamed Sanei

**Affiliations:** ^1^Chemical and Biological Engineering, University of British Columbia, Vancouver, BC, Canada; ^2^Department of Microbiology and Immunology, University of British Columbia, Vancouver, BC, Canada; ^3^NatureWorks Remediation Corporation, Rossland, BC, Canada; ^4^Geological Survey of Canada, Calgary, AB, Canada; ^5^Center for Energy Technologies (CET), AU-Herning, Aarhus University, Herning, Denmark; ^6^Department of Geoscience, University of Calgary, Calgary, AB, Canada

**Keywords:** biochemical reactor, microbial ecology, organic matter degradation, metals, water treatment, bioremediation, sequencing, Rock-Eval-6

## Abstract

Sulfidogenic biochemical reactors (BCRs) for metal removal that use complex organic carbon have been shown to be effective in laboratory studies, but their performance in the field is highly variable. Successful operation depends on the types of microorganisms supported by the organic matrix, and factors affecting the community composition are unknown. A molecular survey of a field-based BCR that had been removing zinc and arsenic for over 6 years revealed that the microbial community was dominated by methanogens related to *Methanocorpusculum* sp. and *Methanosarcina* sp., which co-occurred with *Bacteroidetes* environmental groups, such as Vadin HA17, in places where the organic matter was more degraded. The metabolic potential for organic matter decomposition by *Ruminococcaceae* was prevalent in samples with more pyrolyzable carbon. *Rhodobium-* and *Hyphomicrobium*-related genera within the *Rhizobiales* order that have the metabolic potential for dark hydrogen fermentation and methylotrophy, and unclassified *Comamonadaceae* were the dominant *Proteobacteria*. The unclassified environmental group Sh765B-TzT-29 was an important *Delta*-*Proteobacteria* group in this BCR that co-occurred with the dominant *Rhizobiales* operational taxonomic units. Organic matter degradation is one driver for shifting the microbial community composition and therefore possibly the performance of these bioreactors over time.

## Introduction

Using passive or semi-passive microbial reactors to remove metals from mine or metallurgical waste seepage is very attractive due to the vast repertoire of microbial metabolic responses to metals (Gadd, 2010). Both direct and indirect mechanisms make remediation of metal contaminated water successful. Bioreactors are provided with an organic-rich material, usually a waste product from forestry, pulp and paper, agriculture, or food industries that provides a supply of electron donors to fuel microbial consortia (Lindsay et al., 2008, 2011; Mattes et al., 2011; Schmidtova and Baldwin, 2011). Microbial activity consumes oxygen to create anaerobic and reducing conditions under which several microbial processes, as well as favorable geochemical conditions, occur that promote metal immobilization: fermentative organisms provide electron donors from decomposing complex organic matter; sulfate- and metal-reducing microbes use these electron donors to produce products that lead to metal immobilization (Jalali and Baldwin, 2000; Stolz et al., 2006; Khoshnoodi et al., 2013); membrane-bound metal transporters assimilate metals into microbial cells where they can be methylated and volatilized, accumulated, or reduced to other redox states (Zhang and Frankenberger, 2000; Bentley and Chasteen, 2002; Amoozegar et al., 2012); extrapolymeric substances and cell walls possess binding moieties for metal ions that act as nucleation sites for biosorption and precipitation (Mullen et al., 1989; Jalali and Baldwin, 2000; French et al., 2013). Despite this myriad of avenues for metal transformation and immobilization, biochemical reactors (BCRs) for treatment of mining-influenced water at industrial sites are often beset with sub-optimal performance or even completely fail to reliably meet water quality objectives.

The type of nutrient source used is key and much previous work has taken place to compare different types and combinations of waste materials, such as manure, compost, wood, sawdust, silage, leaf litter, hay, and pulp and paper biosolids, for example, for their efficacy at supporting sulfate-reducing bacteria and removing metals (Waybrant et al., 1998; Zagury et al., 2006; Lindsay et al., 2008; Neculita and Zagury, 2008; Schmidtova and Baldwin, 2011). Attempts in these studies to correlate properties of the organic material such as their carbon and nitrogen content or amount of easily available or more recalcitrant material to sulfate-reduction or metal removal rate have been inconsistent. Most tests using these materials were carried out for only short durations (days, months) and very little is known about how rapidly they would decompose when used in actual BCRs that are expected to operate for years on end. The long-term efficacy of carbon sources used in these bioreactors has been rarely studied.

Despite their perceived role in metal removal mechanisms, the microbial communities supported by these organic materials in BCRs have seldom been characterized. Operators can only speculate on what the reasons for poor operation could be when these systems are not performing well. More work has been done on microbes in so-called active treatment processes, where defined-carbon sources are used in tank-based bioreactors (Dar et al., 2007). Only recently have attempts been made to characterize the much more diverse microbial communities of complex carbon source BCRs (Hiibel et al., 2008), and the importance of the starting microbial community was highlighted as one determinant for successful performance (Pereyra et al., 2008). To our knowledge, there are no studies correlating microbial community composition to organic matter characteristics in field-based metal-removing BCRs. As the organic material degrades over time, the supply of electron donors may change as the more degradable material becomes depleted. This is bound to impact the microbial community composition, which might contribute to different processes taking place in the bioreactor, some of which could compromise reactor performance. In this work, we characterize the microbial community composition in a BCR treating metallurgical waste landfill seepage that had been operating continuously for 6 years since being rebuilt with fresh pulp and paper mill biosolids as the nutrient source. To capture the relative abundance and diversity of the microbial community, samples were taken from the BCR at different spatial locations and at different times of the year. Two molecular methods were employed based on long reads of the small subunit ribosomal ribonucleic acid sequences (SSU rRNA) for high fidelity and high-throughput sequencing of smaller SSU rRNA fragments for greater depth. Microbial community composition correlation with environmental parameters and organic matter properties was evaluated.

## Materials and Methods

### Site characterization and sample collection

**Figure 1:**
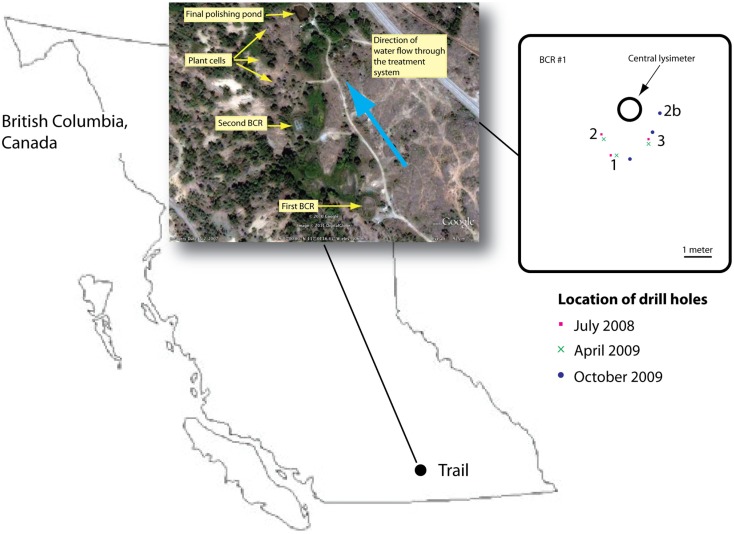
**Location of the BCR in British Columbia, Canada**. Inset provides an aerial view of the whole treatment train according to Google Earth. Second inset is a map of the first BCR that was sampled in this study showing the locations of the drill holes. Drill hole location was chosen so as to avoid damaging internal baffles.

The BCR was located in British Columbia, Canada at 49.115763°N, −117.737118°E, and was the first in a series of sub-surface and surface flow wetlands (Mattes et al., 2011; Figure 1). Influent entered at the bottom of the BCR and flowed upwards through the reactive matrix of pulp and paper biosolids, manure, sand, and crushed pea-sized limestone (Figure S1 in Supplementary Material). Samples were removed during three different seasons in July 2008, April 2009, and October 2009. Saturated material was accessed by drilling three boreholes each time through a soil cover. Cores were removed inside 2-cm internal diameter poly vinyl chloride pipes, which were capped immediately and frozen using liquid nitrogen. In the laboratory, cores were sectioned into 5-cm intervals while still frozen and each section was homogenized by grinding under liquid nitrogen. Homogenized sections were stored at −80°C until DNA extraction.

### DNA extraction and preparation of clone libraries and pyrotag libraries

Aliquots from each homogenized 5-cm interval for all depths and cores were used for extraction of DNA using methods described in detail in the Supplemental Material. Clone libraries for the middle section (20–30 cm depth) of each of the nine cores were produced for Archaea and bacteria using primers targeting the archaeal domain A4F (5′-TCCGGTTGATCCTGCCRG) and U1492R (5′-GGTTACCTTGTTACGACTT) and the bacterial domain B27F (5′-AGAGTTTGATCCTGGCTCAG) and U1492R (5′-GGTTACCTTAGTTACGACTT), respectively, using gradient PCR methods described in detail in the Supplemental Material. Cloned inserts were sequenced bi-directionally with M13F (5′-GTAAAACGACGGCCAG) and M13R (5′-CAGGAAACAGCTATGAC) primers at the Michael Smith Genome Sciences Centre (Vancouver, BC, Canada). Sequences were assembled and checked for chimeras as described in the Supplemental Material.

Different aliquots of the homogenized samples were used for the pyrotag libraries. The DNA was extracted using the Power Soil DNA isolation Kit (MoBio Laboratories Incorporation, Carlsbad, CA, USA, Cat No:12888-100) following the manufacturer’s instructions. DNA from adjacent 5-cm intervals was combined to yield 38 samples from all cores and all depths that were used for PCR amplification of the V6–V8 variable SSU rRNA region: 926f (5′ AAACTYAAAKGAATTGRCGG 3′) and 1392r (5′ ACGGGCGGTGTGTRC 3′) as described in detail in the Supplemental Material. Purified PCR products were sent to the McGill University Innovation Centre (Montréal, QC, Canada) for pyrosequencing using a Roche GS-FLX Titanium Series sequencer. Pyrotag reads were filtered using the following quality control criteria: minimum length 200 bp, no ambiguous base reads, no missing quality scores, mean quality score greater than 25, no more than six nucleotide length homopolymer runs, and no mismatches in reverse primer. Raw pyrotag sequences were deposited in the sequence read archive of the National Center for Biotechnology Information (NCBI) under project PRJNA239169 (Pruitt et al., 2009).

### Bioinformatics and statistics

The Mothur pipeline (Schloss et al., 2009) was executed for clustering the Archaeal clone library sequences into operational taxonomic units (OTUs) using a 97% sequence homology cut-off. A representative sequence for each OTU was picked based on the most abundant read in the OTU bin. Only high-quality SSU rRNA clone reads were used for the analysis and the representative sequences for each OTU were deposited in the NCBI database (Pruitt et al., 2009) (KM213856–KM213864). Qiime (Caporaso et al., 2010) was used to cluster the bacteria clone library sequences and pyrotag reads into OTUs using the methods cd-hit (Li and Godzik, 2006) and usearch (Edgar, 2010), respectively, with 90, 94, or 97% homology cut-offs. Each OTU was assigned a taxonomic classification using BLASTN of the representative sequences to the Silva version 111 curated reference dataset (Quast et al., 2013). Representative sequences for the bacteria clone library 97% OTUs were deposited in the NCBI database (KM250907–KM251172). Phylogenetic trees were constructed using MUSCLE version 3.8.31 for alignment (Edgar, 2004) and the maximum likelihood method for tree building [PHYML (Guindon et al., 2009) with the nucleotide substitution model HKY85 (Hasegawa et al., 1985) and 100 bootstraps]. Sequences for closely related cultured species and environmental clones were found by BLASTN to the NCBI refseq and nucleotide databases (Pruitt et al., 2009), respectively.

Microbial community compositions were compared using two statistical approaches: (1) weighted UniFrac (Lozupone and Knight, 2005) based on phylogenetic distances with principal coordinate analysis (PCoA) using non-metric multidimensional scaling (NMDS) through the R phyloseq wrapper version 1.6.1 (McMurdie and Holmes, 2013) and (2) Bray–Curtis dissimilarity (Jones et al., 2009) visualized on a detrended correspondence analysis (DCA) PCoA diagram produced with the R package vegan version 2.0-10. The latter results were fit to a linear model containing the Rock-Eval-6 variables to test for statistically significant correlations using R (vegan). Co-occurrence of taxonomic groups (94% homology cut-off OTUs) was assessed using Pearson’s correlation of pair-wise OTU read counts. Associations greater than 0.80 correlation and *p*-value less than 0.05 were visualized on a network diagram produced with the R package igraph through the wrapper phyloseq (McMurdie and Holmes, 2013).

### Chemical analysis

Influent and effluent metal and sulfate concentrations were measured every 2 weeks by the operators using inductively coupled mass spectroscopy (ICP-MS) in an industrial metallurgical laboratory. At the time of sampling, pore water pH, temperature, dissolved oxygen, and oxidation–reduction potential were measured using a 6600V2 sonde with appropriate probes (YSI Inc., Yellow Springs, OH, USA). Pore water nitrite, nitrate, ammonia, total phosphorous, sulfide, and Fe^2+^ were measured *in situ* using field kits (Hach company, Loveland, CO, USA; CHEMetrics, Midland, VA, USA). Samples were preserved using 0.3% zinc acetate for sulfate analysis later in the laboratory using standard method $4500{\mathrm{-SO}}_{4}^{2-}$ (Eaton et al., 2005). The degree of organic matter degradation was deduced using the pyrolysis technique Rock-Eval-6 (Behar et al., 2001) on 33 samples. This technique characterizes the organic matter in terms of several fractions: S1 refers to the light hydrocarbons (HCs) that pyrolyze first with units of milligram HC per milligram total organic carbon (TOC); S2 contains organic matter that is removed next. Pyrolyzable carbon (PC) is the sum of S1 and S2 plus CO and CO_2_ evolved during pyrolysis. Residual carbon (RC) refers to the remaining organic matter that is combusted. Additionally, mineral carbon (MINC) is quantified also in the Rock-Eval-6 process. More details and example Rock-Eval-6 spectra are given in the Supplementary Material.

## Results

### BCR performance and environmental conditions

**Figure 2:**
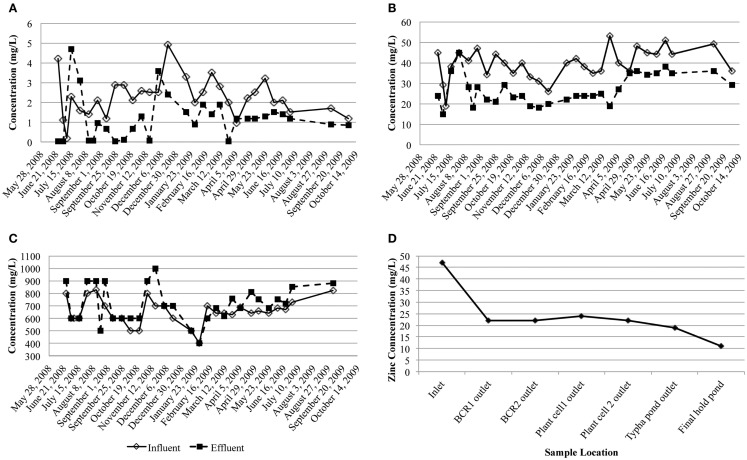
**Influent and effluent concentrations of dissolved (A) arsenic, (B) zinc, and (C) sulfate (milligram per liter) over the study period**. **(D)** Dissolved zinc concentration through the treatment system taking into account the hydraulic retention time of each step.

Dissolved arsenic, zinc, and sulfate influent concentrations averaged 2.3 (1.0) mg/L, 40 (8) mg/L, and 650 (100) mg/L, respectively (Figure 2). On average, 47.8% arsenic and 34.2% zinc were removed but the average sulfate concentration increased by 9.0%. Influent flow rates averaged 477.8 m^3^/month except for August 2008 when an exceptional amount of seepage (1,703 m^3^) entered the system. Based on estimated hydraulic retention times (~4 weeks) through each of the stages in the whole treatment system, most of the zinc was removed in the first BCR (Figure 2D). During sampling, conditions inside the BCR were reducing (ORP less than −112 mV), with circum-neutral pH (5.6–7.5) and low amounts of dissolved oxygen (0–1.5 mg/L; Table 1). Sulfide was detected in the pore water in July only (1.1–3.5 mg/L). Sulfate concentrations in the pore water varied from 80 to 600 mg/L (Table 1). Dissolved oxygen in the pore water was lowest (below the detection limit) in July, when temperatures were highest (17.6°C average). The coldest temperatures encountered were in April (7.1°C average). There were no significant seasonal variations in pH or ORP. Although there were no significant differences in metal removal with season according to the influent and effluent data collected by the operator (Figure 2), the pore water chemistry differed. The presence of sulfide and lower concentrations of sulfate and metals during July 2008 than the other months suggested that the bioreactor was more active in terms of sulfate-reduction when the temperatures were warmer and the DO lower. Total phosphorous concentrations in April were lower than those measured in July and October, which might have been a nutrient limitation at that time.

**Table 1:** **Pore water chemistry for each of the boreholes at the time of sampling**.

Date	July 2008			April 2009			October 2009		

Hole number	1	2	3	1	2	3	1	2	3
**Pore water parameters measured** ***in situ***
Dissolved oxygen (mg/L)	0.0	0.0	0.0	1.5	1.1	0.7	1.2	1.3	1.3
pH	5.6	5.6	6.9	6.9	6.9	7.0	6.7	6.2	7.5
Oxidation/reduction potential (mV)	−133	−112	−238	na	na	na	−118	−244	−130
Temperature (°C)	17.6	15.9	19.4	7.6	6.5	7.1	11.2	11.5	11.2
**Dissolved metals (mg/l)**
As	0.19	0.06	0.03	0.94^a^	0.94	0.94	0.14	0.21	0.51
Cd	0.06	0.02	0.01	0.002	0.002	0.002	0.0006	0.0012	0.055
Mn	78	110	3.9	7.6	7.6	7.6	1.5	22	5.7
Fe	740	910	50	7.6	7.6	7.6	1	4.25	0.3
Sb	0.01	0.01	0.01	0.014	0.014	0.014	0.0028	0.002	0.0011
Zn	6.1	1.3	0.52	0.058	0.058	0.058	0.045	0.093	0.28
**Nutrients measured with chemets and hach kits (mg/l)**									
Sulfide	1.1	2.6	3.45	0	0	0	0	0	0
Total phosphorous	21	20	30	0.5	0.5	0.5	24	14	6.3
Sulfate	200	80	150	470	470	470	600	150	270
Nitrite/nitrate	na/5.2	na	na	0.015/1.3	0.015/1.3	0.015/1.3	0.023/5.5	0.109/5.5	0.01/1.5
Ammonium-N	460	340	490	61	61	61	60	76	36
**Core solids chemistry**
Total C (%)	9.39	10.65	6.23	NSS	10.6	18.3	18.3	7.12	3.32
Total S (%)	0.24	0.19	0.11	NSS	0.31	0.27	0.27	0.15	0.09
As (ppm)	2.7	2.9	1.8	>250	16.6	18.9	45.8	9.3	18.9
Cd (ppm)	1.7	2	1.6	59.4	2.8	2.8	4.7	1.1	3.7
Zn (ppm)	108	122	18.6	1735	180	177	705	127	380
Loss on ignition (%; 1,000°C)	25	29.5	118	NSS	24.8	30.1	38.7	23.1	8.49

*na, not available; NSS, not enough sample for the analysis*.

*When attempting to measure the ORP in April, two ORP probes were used. The ORP value continued to drop without stabilizing even after over an hour of being immersed in the pore water or lysimeter water*.

*^a^There was insufficient water for chemical analysis from the bore holes, therefore water from the central lysimeter, which was located in close proximity to the boreholes, at the same depth as the bore holes was analyzed instead*.

### Solids chemistry

Organic matter (OM) characteristics were determined so as to estimate the degree of degradability of the matrix at the time of sampling. Rock-Eval-6 is a pyrolysis method used to study the composition of organic material with different maturities; most often coal and other HCs, but also organic matter in soils and sediments (Behar et al., 2001). The TOC measured in the Rock-Eval-6 analysis in the BCR core samples averaged 4.63 ± 2.45 wt% compared with 11.1 ± 0.9 wt% in fresh matrix of a similar composition to that in the BCR. The bulk of the pyrolyzable compounds were liberated under the S2 peak (Table 2), which was typically broad in contrast to the narrower S1 peak (Sebag et al., 2006; Figure S2 in Supplementary Material). Both peaks contained shoulders indicating mixtures of compounds. Pyrolyzable material under the S1 peak was roughly half that of S2 in the BCR samples. The average ratio of the S1 to S2 fraction was 0.45 compared to 0.59 for the fresh biosolids. Less than half of the TOC was as the PC fraction, which was lower than that in a fresh pulp mill biosolids sample containing sand and limestone in the same ratios as was in the original BCR mixture.

**Table 2:** **Rock-Eval results for the core samples**.

Sample ID	Month collected	Hole no.	Depth	S1/TOC	S2/TOC	PC/TOC	RC/TOC	MINC/TOC	*T*_max_	*T*_peak_
			cm	(mg-HC/TOC)		(fraction)			(°C)	
TC1	July	1	0–10	166	306	0.447	0.553	1.4	316	354
TC2	July	1	10–20	155	310	0.446	0.554	0.9	324	362
TC3	July	1	20–30	154	316	0.448	0.552	0.6	324	362
TC4	July	1	30–40	104	268	0.394	0.606	5.6	330	368
TC5	July	1	40–50	115	273	0.373	0.627	0.3	327	365
TC6	July	2	0–10	151	289	0.414	0.586	1.0	321	359
TC7	July	1	10–20	149	276	0.399	0.601	1.5	318	356
TC8	July	2	20–30	147	286	0.403	0.597	1.2	323	361
TC9	July	2	30–40	138	287	0.395	0.605	2.1	313	351
TC10	July	2	40–50	134	282	0.389	0.611	0.6	324	362
TC11	July	3	0–10	135	294	0.398	0.602	0.5	315	353
TC12	July	3	10–20	130	293	0.391	0.609	0.3	314	352
TC13	July	3	20–30	116	288	0.382	0.618	1.0	314	352
TC14	July	3	30–40	121	275	0.375	0.625	1.1	313	351
TC15	July	3	40–50	128	285	0.385	0.615	1.5	316	354
TC16	April	1	0–10	90	255	0.346	0.654	1.1	314	352
TC17	April	1	10–20	108	243	0.338	0.662	1.3	317	355
TC18	April	1	20–30	127	260	0.363	0.638	0.5	316	354
TC19	April	1	30–40	126	277	0.384	0.616	1.5	312	350
TC22	April	2	20–30	164	307	0.448	0.552	0.7	320	358
TC24	April	3	0–10	148	307	0.422	0.578	0.6	319	357
TC25	April	3	10–20	143	288	0.406	0.594	1.4	331	369
TC26	April	3	20–30	133	266	0.366	0.634	0.4	316	354
TC27	April	3	30–40	136	273	0.378	0.622	0.5	329	367
TC28	October	1	0–10	104	289	0.379	0.621	6.6	314	352
TC29	October	1	10–20	106	287	0.383	0.617	4.5	315	353
TC30	October	1	20–30	128	329	0.421	0.579	0.1	338	376
TC31	October	2	0–10	127	317	0.432	0.568	0.6	319	357
TC32	October	2	10–20	148	307	0.435	0.565	0.5	321	359
TC33	October	2	20–30	111	324	0.424	0.576	1.8	327	365
TC34	October	2	30–40	154	314	0.444	0.556	0.5	319	357
TC35	October	2	40–50	117	321	0.407	0.593	0.9	319	357
TC38	October	3	20–30	102	313	0.391	0.609	0.6	321	359
			Min.	90	243	0.338	0.552	0.1	312	350
			Max.	166	329	0.448	0.662	6.6	338	376
			Ave.	131	291	0.400	0.600	1.3	320	358
			Std. dev.	20	21	0.030	0.030	1.5	6	6

### Overall microbial diversity

**Figure 3:**
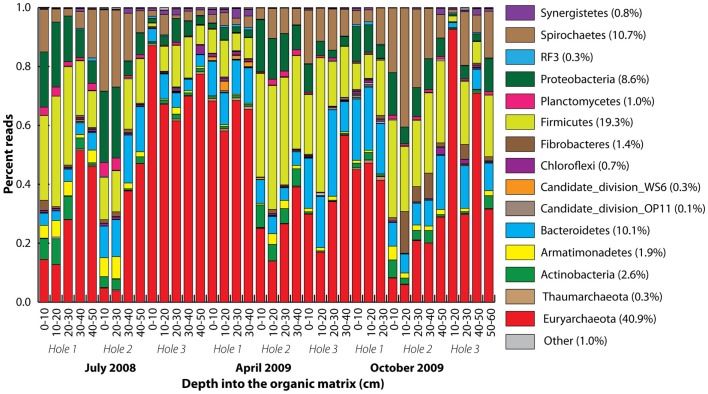
**Phylum-level taxonomic summary of 90% homology cut-off pyrotag reads**. Phyla stacked according to color from the bottom to the top, starting with *Euryarchaeota*. Rare phyla were all colored gray (other). Vertical axis represents percent reads, and the horizontal axis depth (centimeter) into the organic matrix.

A total of 416 Archaea and 730 bacteria clone library SSU rRNA sequences were obtained yielding 9 and 268, respectively, 97% homology cut-off OTUs. After quality control, 208,483 pyrotag reads were obtained, with the number of reads per sample varying from 1,083 to 10,356. The 1,667 97% homology pyrotag OTUs were classified into a total of 37 phyla within the Archaea (2), Bacteria (32), and Eukaryota (3) domains. The pyrotag reads revealed that 15 phyla dominated with *Euryarchaeota*-related sequences the most abundant (Figure 3). Proportional representation of the phyla varied across the samples. In some cases (Holes 1 and 3 in July and Hole 2 in October), the percentage of *Euryarchaeota*-related reads increased with depth, but this was not statistically significant overall. There were no statistically significant differences between the microbial community compositions of different seasons, with the same dominant 14 phyla represented throughout.

**Figure 4:**
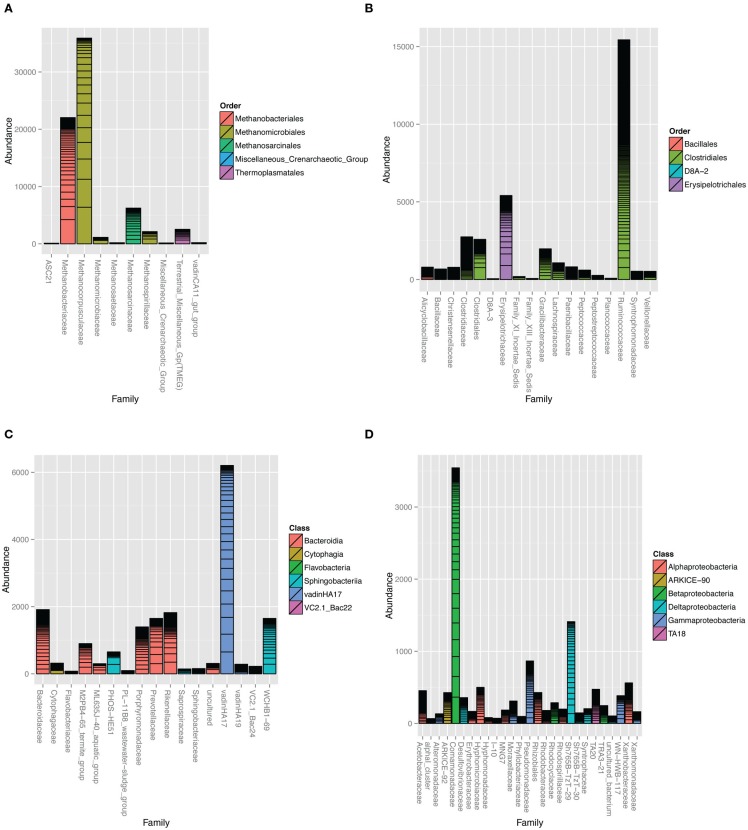
**Taxonomic summaries of read counts assigned to different families in the (A) *Archaea* domain and (B) *Firmicutes*, (C) *Bacteroidetes*, and (D) *Proteobacteria* phyla**. Bars represent 90% homology cut-off OTUs stacked according to their abundance. Family bars are colored according to order. Vertical axis represents the number of pyrotag reads assigned to each family-level OTU. Barplots were constructed with the phyloseq (http://joey711.github.io/phyloseq/) suite of packages and dependencies in R version 2.15.2.

The Archaea clone SSU rRNA sequences formed five major clades (Figure S3 in Supplementary Material). The majority of sequences were classified as Class II methanogens in the orders *Methanomicrobiales* and *Methanosarcinales* closely related to *Methanocorpusculum labreanum*, *M. parvum*, *Methanosarcina vacuolata*, and *M. barkeri*. Close environmental relatives came from anaerobic digesters (Godon et al., 1997) and HC environments, such as tar pits, biodegraded oil reservoirs, and coal beds (Figure S3 in Supplementary Material; Zhao et al., 1989; Grabowski et al., 2005a; Strapoc et al., 2008). Pyrotag sequences revealed that family-level *Methanocorpusculaceae*-related OTUs dominated the overall microbial community (Figure 4A) with *Methanocorpusculum* as the most highly represented genus (Figure S4 in Supplementary Material). Unclassified *Methanobacteriaceae* were highly prevalent although none of the clone library sequences were classified in this family.

Most bacteria were classified into the phyla *Firmicutes*, *Bacteroidetes*, *Proteobacteria*, and *Spirochetes* according to both the clone library and pyrotags (Figure 3; Figure S5 in Supplementary Material). Additionally, Candidate Division environmental groups were present, such as WS6 and OP11, within which no species have been cultured or characterized. These are widely represented in many environments including bioreactor sludge as well as soils (Dinis et al., 2011; Youssef et al., 2011). Apart from the *Euryarchaeota*, the most highly represented genus-level OTUs were *Treponema*-related (in the *Spirochetes* phylum; Figure S5 in Supplementary Material).

Bacteria associated with organic matter digestion, such as *Ruminococcaceae* and *Erysipelotrichaceae* were highly represented in the *Firmicutes* phylum (Figure 4B; Figure S6 in Supplementary Material). In particular, one genus-level OTU classified as *Erysipelotrichaceae* was the seventh most prevalent (Figure S4 in Supplementary Material). Clone library sequences related to cellulose degrading and fermenting *Clostridium* species were well represented.

Environmental groups were highly represented in the *Bacteroidetes* phylum (Figure 4C; Figure S7 in Supplementary Material), especially VadinHA17, WCHB1-69, and U29-B03 within the *Rikenellaceae* family. The pyrotags and clones revealed other environmental groups such as the M2PB4-65_termite_group (Hongoh et al., 2005) and ML635J-40_aquatic_group from Mono Lake (Humayoun et al., 2003) and PHOS-HE51 (Dabert et al., 2001).

Microbes classified in the *Comamonadaceae* family within the *Beta-Proteobacteria*, *Rhizobiales* order within *Alpha-Proteobacteria* and a *Delta-Proteobacteria* environmental group Sh765B-TzT-29 were the most important *Proteobacteria* (Figure 4D; Figure S8 in Supplementary Material). Two particularly predominant OTUs classified as *Rhodobium*/*Hyphomicrobium* (Figures S4 and S8 in Supplementary Material). At the time of writing, there were 44 environmental clones in the NCBI nucleotide database that were closely related to the predominant BCR *Rhodobium*-classified clone (>97% identity). Of these, 17 came from wetland soils, 11 from natural or agriculture soils, 12 from contaminated soils (mining, radioactive waste, and HCs) and 4 from marine and cold-water sediments. OTUs classified in the environmental group Sh765B-TzT-29 were phylogenetically distinct from all the other *Proteobacteria* sequences (Figure S8 in Supplementary Material). Putative sulfate-reducing bacteria were rare, with some pyrotag sequences classified as *Desulfovibrio* and *Desulfobulbus* genera (Table S1 in Supplementary Material). *Clostridia*-related OTUs classified as *Desulfosporosinus* and *Desulfotomaculum* were present also (Table S1 in Supplementary Material).

*Armatimonadetes*-related reads were predominant (Figures S4 and S9 in Supplementary Material). At the time of writing, there were no other environmental clones more than 94% homologous to the bioreactor *Armatimonadetes*-related clone. *Fibrobacteres* (*Fibrobacter*), *Planctomycetes* (AKAU3564 sediment group), and *Synergistetes* (*Synergistaceae*) were three other phyla well represented in the bioreactor.

### Correlation of microbial community with organic matter characteristics

**Figure 5:**
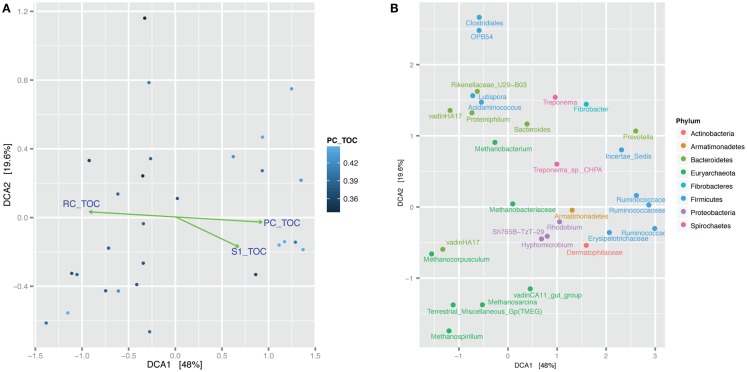
**Bray–Curtis dissimilarity detrended correspondence analysis (DCA) comparison of microbial communities in the samples**. **(A)** Samples are shaded according to the fraction of pyrolyzable carbon to total organic carbon (PC/TOC). Green lines and arrows indicate statistically significant correlation of Bray–Curtis dissimilarity with fraction pyrolyzable carbon to total organic carbon. Samples containing more PC/TOC cluster on the right-hand side and those will more residual carbon (RC/TOC) cluster on the left-hand side. **(B)** Same analysis showing clustering of the top 30 most abundant OTUs with samples. OTUs (dots) labeled according to their lowest taxonomic assignment and colored according to Phylum.

The 94% homology cut-off OTUs were rarefied to 4995 reads each per sample and then trimmed to exclude low diversity OTUs that were represented by fewer than three read counts and present in less than 20% of the samples. Comparison of sample microbial communities using weighted UniFrac analysis revealed two broad groups that appeared to cluster according to their ratio of PC to TOC (PC/TOC) (Figure S10 in Supplementary Material). UniFrac distances could not be used for correlation with the organic matter characteristics and Bray–Curtis dissimilarity indices based on OTU read counts were used instead (Figure 5A). Using a general linear model to fit the Bray–Curtis dissimilarities to the organic matter characteristics, a statistically significant correlation was revealed with the fraction of PC or RC (*p*-values <0.001), and to a lesser extent the S1 fraction (*p*-value <0.5; Table 3). No statistically significant correlations were found between the microbial community composition and any of the pore water chemistry parameters (Table 1).

**Table 3:** **Statistical results of the linear fit of microbial community dissimilarities according to Bray–Curtis indices to organic matter characteristics as determined by Rock-Eval-6 variables in Table 2**.

Variable	DCA1	DCA2	*R*^2^	*p*-Value	Significance
S1/TOC	0.697	−0.717	0.240	0.024	^a^
S2/TOC	0.826	−0.564	0.122	0.201	
PC/TOC	0.726	−0.688	0.343	0.005	^b^
RC/TOC	−0.726	0.698	0.343	0.005	^b^
MINC/TOC	−0.100	−0.995	0.087	0.345	

Significance codes:

^a^<0.05;

*^b^<0.01*.

**p*-Value based on 999 permutations*.

Prevalent OTUs and their taxonomic classifications were layered on to the same DCA plot for further interrogation of the phylogeny associated with more pyrolyzable or RC (Figure 5B; Figure S11 in Supplementary Material). *Methanocorpusculum* and VadinHA17 clustered together with samples containing more RC, whereas *Ruminococcaceae* were more likely to be found with higher amounts of PC. *Proteobacteria* OTUs such as those classified as *Rhodobium, Hyphomicrobium*, and Sh765B-TzT-29 appeared more so in the samples with higher PC content.

Correlation of microbial community structure with organic matter characteristics was tested for each of the months separately (Figure S12 in Supplementary Material). Statistically significant fits of microbial community composition with Rock-Eval-6 variables PC and, to a lesser extent, S1 were obtained for the July and October samples, but not with the April samples. The prevalent OTUs clustered similarly in the July and October samples.

**Figure 6:**
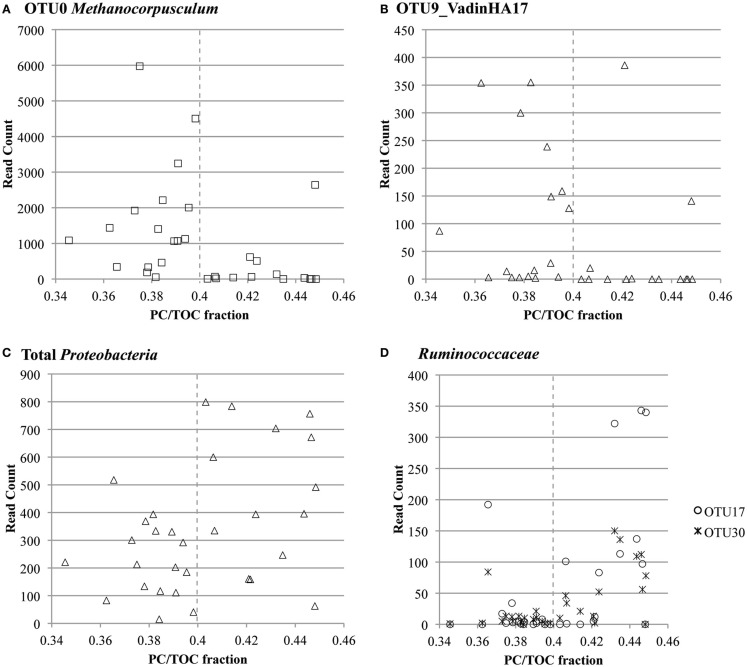
**OTU read count versus ratio of pyrolyzable carbon to total organic carbon (PC/TOC) for select highly prevalent taxa**: **(A)** OTU0 classified as *Methanocorpusculum* related, **(B)** OTU9 classified in the environmental group VadinHA17, **(C)** all OTUs classified in the *Proteobacteria* phylum and **(D)** OTUs 17 and 30 classified in the *Ruminococcaceae* family.

Co-occurrence of taxa in these specific clusters was confirmed with Pearson’s correlation analysis (Figure S13 in Supplementary Material). Prevalent *Proteobacteria-*, *Firmicutes-* (*Ruminococcaceae*), and *Actinobacteria*-related OTUs co-occurred with each other (Pearson’s correlation coefficient >0.8, *p*-value <0.01). OTU read counts plotted versus PC/TOC fraction confirmed prevalence of the most abundant *Methanocorpusculum*- and VadinHA17-related OTUs with the samples containing more recalcitrant organic matter, and the greater prevalence of *Proteobacteria* and *Ruminococcaceae* where there was more PC (Figure 6).

## Discussion

Important sustainable mechanisms for metal removal in BCRs are microbially driven. Detailed analysis of the microbial communities in these systems can be done using molecular surveys of the SSU rRNA gene. This reveals insight into the metabolic potential inside the bioreactor, and correlation of community composition with environmental and operational parameters provides valuable information on possible causes of metabolic shifts that may impact bioreactor performance. Reactors of this type are not perfectly mixed and environmental conditions inside the reactor might be heterogeneous. Additionally, they operate all year round and temporal shifts might influence the metabolic potential. For these reasons, samples taken across spatial and temporal gradients were needed to characterize the microbial community. The affordability and high-throughput of next generation sequencing enabled us to do this for the first time for a bioreactor of this type. Sequencing revealed consistency in terms of the taxonomic groups represented over depth and season, with some variations in their proportional representation. In particular, some niches inside the BCR were dominated by methanogens. This was not expected. High concentrations of sulfate were measured in the bioreactor influent and in the pore water during sampling. If sulfate is non-limiting, sulfate-reducers are expected to outcompete methanogens due to thermodynamic favorability for sulfate-reduction (Raskin et al., 1996). Although these observations have held true for active bioreactors receiving defined-carbon sources (Dar et al., 2008), co-occurrence of methanogens and SRB has been observed in complex carbon bioreactors (Logan et al., 2003; Rezadehbashi and Baldwin, 2014). It is not uncommon for sulfate-reducers to form only a small portion of the bacterial community in complex organic matter BCRs, since most microbes present in these bioreactors have the metabolic potential for organic matter degradation (Schmidtova and Baldwin, 2011). Even in the case of a defined-carbon source bioreactor, sulfate-reducers comprised less than 10% of all bacteria (Kaksonen et al., 2006). Very few studies are available on the microbial community composition of metal-treating complex carbon BCRs that have been operating in the field. Hiibel et al. (2008) surveyed the bacterial community of two pilot-scale field-based bioreactors treating acid mine drainage using 16S rRNA clone libraries, but they did not include *Archaea* in their survey. Unpublished work from our laboratory indicates that in some field-based BCRs, sulfate-reducers are more abundant than methanogens (Rezadehbashi and Baldwin, 2014).

Although sulfate-reducers were rare at the time of this study, and restricted to only a few lineages, there was evidence for sulfide reduction: sulfide was detected in the BCR pore water during the summer. The chemical environment of the bioreactor was not toxic for sulfate-reducers as fresh organic materials of different types suspended in the bioreactor pore water were able to support a wide diversity of sulfate-reducing bacteria (Schmidtova and Baldwin, 2011). There is evidence that sulfate-reducers were more active in the BCR in the past. A mineralogical survey of the same BCR core samples as were used in this study revealed that As and Zn were present mostly as sulfides inside the bioreactor (Khoshnoodi et al., 2013). Data collected by the operators from previous years shows that sulfate was being reduced through the system (Mattes et al., 2011). According to influent and effluent chemical analysis performed by the operators, sulfate-reduction was not occurring in the BCR during the study period, thus it might have been the case that this system was shifting from sulfidogenesis to methanogenesis.

A pyrolysis-based technique proved to be useful for characterizing the degree of degradation of organic matter in the BCR. Other studies have used Rock-Eval to characterize soil organic matter. Deconvolution of the S2 peak revealed several overlapping peaks due to different classes of chemical compounds releasing at different temperatures (Sebag et al., 2006; Carrie et al., 2012). *T*_peak_ values can be used to indicate the type of dominant organic matter in the mixture. Cellulose and lignin produce a peak at around 350°C when pyrolyzed as pure forms (Disnar et al., 2003). Thus, in the BCR core samples, the main S2 peak likely was due to pyrolysis of polysaccharides, polypeptides, and lignin. This main peak was more pronounced in the samples containing more PC (Figure S2C in Supplementary Material). Low molecular weight bioploymers such as sugars and lipids that are more biodegradable contribute to the S1 peak (Sebag et al., 2006; Carrie et al., 2012). Statistically significant correlation of microbial community structure with Rock-Eval-6 PC, and to a lesser degree the S1 peak, suggested that the availability of organic matter may have been playing role in shifting of the BCR metabolic potential toward favoring methanogens. Association of methanogens with highly degraded environments was further supported in that close relatives to the BCR methanogens came from highly degraded carbon environments. The BCR was dominated by hydrogenotrophic methanogens (*Methanocorpusculaceae* and *Methanobacteriaceae*), although some acetoclastic groups were also present (i.e., *Methanosarcina* sp.). *Rhodobium*-related species have been found to be involved in dark carbon fermentation, which produces hydrogen (Patel and Kalia, 2013). OTUs in the BCR related to *Rhodobium* species, and *Bacteroidetes*, *Firmicutes*, *Spirochetes*, and *Synergistetes*-related OTUs that clustered with low PC and methanogen-enriched samples, likely include candidates for methanogen syntrophy. Thus, one metabolic cycle in the BCR potentially revolves around hydrogen and methane, which is analogous to what has been reported for highly degraded oil reservoirs where *Rhizobiales* have also been found to be abundant microbes together with methanogens (Zhang et al., 2012). *Rhizobiales* species are capable of arsenic resistance (Lim et al., 2014) and their relatives in the BCR may also have metal resistance pathways. Additionally, *Hyphomicrobium* spp. are methylotrophic (grow on C1 carbon compounds) and their relatives present in the BCR might have been involved in methane metabolism (Costa et al., 2000). The closest cultured relative to the BCR *Hyphomicrobium*-classified OTUs, *H. vulgare*, is prevalent in denitrifying bioreactors fed with methanol. Nitrate was present in the BCR feed water (average of 68.5 ± 14 mg/L in July 2008 and average of 35.8 ± 9 mg/L in April 2009). The availability of methane and nitrate may explain the prevalence of this group in the BCR.

Many bacteria classified in the phylum *Proteobacteria* are important for metal biotransformation. Although the BCR *Comamonadaceae* OTUs were unclassified, some were related to genera associated with anaerobic arsenite oxidation (e.g., *Acidovorax*; Huang et al., 2012). Sulfate-reducers detected in the BCR were classified in taxa (*Desulfovibrionaceae*, *Desulfosporosinus*, and *Desulfotomaculum*) also found in other high metal environments (Lee et al., 2009; Sánchez-Andrea et al., 2011). However, the most prevalent *Delta-Proteobacteria* found in the BCR, the environmental group Sh765B-TzT-29, had not been associated with these types of bioreactors until this study. This group was first assigned to *Geobacteraceae* as a cluster of heavy metal-associated microbes, and recently has been linked tentatively to methane oxidation in culturing experiments (Siegert et al., 2011). It is unknown if Sh765B-TzT-29 plays any role in the sulfur cycle.

The BCR contained other novel taxonomic groups that were predominant but whose role is unknown, such as those classified as *Erysipelotrichaceae*. Genera within these families are predominant gut bacteria, some of which are associated with high fat diets (Zhang et al., 2010). Members of the *Bacteroidetes* environmental groups VadinHA17, WCHB1-69, and U29-B03, also predominant in the BCR, are found in environments undergoing complex carbon degradation. Clone VadinHA17 came from an anaerobic digester treating winery wastewater (NCBI accession number U81712). The first member of WCHB1-69 was a clone found in an aquifer contaminated with HCs and chlorinated-solvents that was undergoing intrinsic bioremediation (NCBI accession number AF050545; Dojka et al., 1998). Most sequences classified in U29-B03 are from rumen environments according to the Silva SSU r117 database. Other *Bacteroidetes*-related OTUs were closely related to cultured species that produce acetate, H_2_, CO_2_, and possibly volatile sulfur compounds using elemental sulfur or nitrate as electron acceptors (Grabowski et al., 2005b; Krespi et al., 2006).

Overall, the microbial community in the BCR consisted of highly specialized groups closely related to other organisms associated with metal-rich environments as well as highly degraded HC reservoirs experiencing terminal carbon degradation by methanogens and possibly microbes within the *Rhizobiales* order. Predominant taxonomic groups revealed metabolic potential for methane production, metal-tolerance, and resistance, and many different types of fermentation including possibly dark fermentation to produce hydrogen. Sulfate-reduction potential according to known SRB taxonomic groups was less represented. Novel groups predominant within the BCR may contain organisms involved in sulfur cycling and metal resistance. The microbial community broadly delineated according to the degree of organic matter degradation with methanogens and putative syntrophs, dominant in more recalcitrant material and *Proteobacteria*, *Actinobacteria*, *Armatimonadetes*, and *Planctomycetes* prevalent in places with more PC.

## Conclusion

This work has contributed to opening the “black box” of bioremediation of metal and sulfate-contaminated water. It was revealed for this particular system that sulfate-reducers, desired for metal sulfide precipitation, were rare and restricted to a few specific genera: *Desulfobulbus*, *Desulfovibrio*, *Desulfosporosinus*, and *Desulfotomaculum*. The bioreactor was dominated by methanogens, with a *Methanocorpusculum*-related OTU most prevalent. Correlation of methanogen-related OTUs with more degraded material suggests that as these types of bioreactors age, methanogens might become more prevalent. Many novel, uncharacterized taxonomic groups were found and suggest that the metabolic potential for organic matter degradation or even metal transformation is much more diverse than previously thought.

## Author Contributions

The manuscript was written through contributions of all authors. All authors have given approval to the final version of the manuscript.

## Conflict of Interest Statement

The authors declare that the research was conducted in the absence of any commercial or financial relationships that could be construed as a potential conflict of interest.

## Supplementary Material

The Supplementary Material for this article can be found online at http://www.frontiersin.org/Journal/10.3389/fbioe.2015.00027/abstract

This includes details of the molecular biology materials and methods, a table of the putative sulfate-reducing bacteria found in the BCR pyrotag library, a table of the highly correlated co-occurring OTUs, a simple cartoon of the BCR, details for the Rock-Eval-6 analysis, phylogenetic trees clone library SSU rRNA 97% homology cut-off OTUs, charts of the relative abundance of the most highly prevalent pyrotag OTUs, UniFrac principal component analysis of the microbial communities, Bray–Curtis dissimilarity detrended correspondence analysis of microbial communities faceted according to Phylum, Bray–Curtis dissimilarity detrended correspondence analysis of samples split according to season and a network of the most highly correlated OTUs.
