# Supplementary material for: The Microbial Community of a Passive Biochemical Reactor Treating Arsenic, Zinc, and Sulfate-Rich Seepage
Source: Front Bioeng Biotechnol. 2015 Mar 6;3:27. doi: 10.3389/fbioe.2015.00027 (PMC4351619; doi:10.3389/fbioe.2015.00027)
Supplement: Supplementary file 1 [file Presentation_1.PDF]

Supplementary Material

The microbial community of a passive biochemical reactor treating arsenic, zinc and sulfate-rich seepage

Susan A. Baldwin[1]*, Maryam Khoshnoodi[1], Maryam Rezadehbashi[1], Marcus Taupp[2], Steven Hallam[2], Al Mattes[3] and Hamed Sanei[4,5,6]

1. Chemical and Biological Engineering, University of British Columbia, 2360 East Mall, Vancouver, BC V6T 1Z3, Canada

2. Department of Microbiology and Immunology, 2350 Health Sciences Mall, University of British Columbia, Vancouver, BC V6T 1Z3, Canada

3. NatureWorks Remediation Corporation, Box 515 Rossland, BC VOG 1YO, Canada

4. Geological Survey of Canada, 3303-33rd Street NW, Calgary, AB, Canada

5. Center for Energy Technologies (CET), AU-Herning, Aarhus University, Denmark

6. Department of Geoscience, University of Calgary, Calgary, AB, Canada

1. Supplementary Materials and Methods

1.1. DNA Extraction for clone libraries

Half a gram of homogenized sample was placed into a 2mL Lysing Matrix E tube (MP BioMedicals, Solon, OH, USA) with 300μL Miller phosphate buffer and 300μL of Miller SDS lysis buffer (Miller et al., 1999). Six hundred microliters of phenol:chloroform:isoamyl-alcohol (25:24:1) was added and the tube inverted several times to aid mixing. The tube was placed in a FastPrep Automated Homogenizer system (MP BioMedicals, Solon, OH, USA) for 45s at 6.5m/s, placed on ice and then centrifuged at 16000g for 5min. Five hundred and sixty microliters of supernatant was transferred to a phase-lock gel tube (MaXtract, Qiagen, Mississauga, ON, Canada), 560μL of chloroform was added and the tube inverted 4 times. Tubes were centrifuged at 10000g for 3min and the top layer transferred to a microcentrifuge tube. Nine hundred microliters of MoBio solution S3 (UltraClean Soil DNA isolation kit, MoBio, Carlsbad, CA, USA) was added to the tube and gently mixed. The mixture was loaded in stages onto a MoBio Spin Filter centrifuged for 30s at 10000g. Four hundred microliters of MoBio solution S4 was added to the filter and the column centrifuged at 10000g for 30s. The flow-through was discarded and the filter was spun again at 10000 g for 1 min. The spin filter was placed in an empty collection tube and 40μL of MoBio Solution 5 was added, followed by incubation for 1min and then centrifugation at 10000g for 30s. Eluted DNA was stored at -20°C.

1.2. Clone library construction and sequencing

Clone libraries for the middle section (20-30cm depth) of each of the nine cores were produced for archaea and bacteria. DNA extracts were amplified using primers targeting the archaeal domain A4F (5'- TCCGGTTGATCCTGCCRG) and U1492R (5'- GGTTACCTTGTTACGACTT) and the bacterial domain B27F (5'- AGAGTTTGATCCTGGCTCAG) and U1492R (5'- GGTTACCTTAGTTACGACTT). For maximal diversity gradient PCR was performed: denaturation for 3min at 95°C; 30 cycles of 95°C for 20s, 48°C-58°C for 20s, 72°C for 2min followed by a final extension of 6min at 72°C. Each 50µl reaction contained 1µl of template DNA, 1.25µl each 10mM forward and reverse primer, 2.5U Taq (Herculase Enhanced DNA Polymerase, Agilent, La Jolla, CA, USA), 5µl 10mM deoxynucleotides, and 30.5µl 10X Herculase PCR Buffer.

The SSU rRNA gene amplicons were pooled followed by visualization on 1% agarose gels in 0.5X TBE and purified using the QIAquick Gel Extraction Kit (Qiagen, Mississauga, ON, Canada). Samples were recovered in 30µl of elution buffer (10mM Tris-Cl pH 8.5). Approximately 4µl of each purified SSU rRNA gene product was cloned into a pCR-Blunt-II-TOPO vector using the Zero Blunt TOPO PCR cloning kit (Invitrogen, Carlsbad, CA, USA) and transformed by chemical transformation into One Shot Mach1 T1 phage-resistant chemically competent Escherichia coli cells (Invitrogen, Carlsbad, CA, USA) and selected on LB agar plates supplemented with 50µg kanamycin/mL and 40µl X-gal (20mg/mL stock). Transformants were transferred to 96-well plates (2 for the bacterial amplicon and one for the archaeal amplicon) containing 180µl LB supplemented

with 50µg kanamycin/mL and 10% glycerol and grown overnight at 37°C prior to storage at −80°C. Cloned inserts were sequenced bi-directionally with M13F (5′-GTAAAACGACGGCCAG) and M13R (5′-CAGGAAACAGCTATGAC) primers at the Michael Smith Genome Sciences Centre (Vancouver, BC, Canada). Sequences were edited manually from traces using Sequencher software V4.9.1 (Gene Codes Corporation, Ann Arbor, MI, USA). The resulting data sets were checked for chimeric sequences using Bellerophon (Huber et al., 2004).

1.3. DNA extraction for pyrotag libraries

Different aliquots of the homogenized samples were used for the pyrotag libraries. The DNA was extracted using the Power Soil DNA isolation Kit (MoBio Laboratories Incorporation, Carlsbad, CA, USA, Cat No:12888-100) following the manufacturer's instructions. DNA from adjacent 5cm intervals was combined to yield 38 samples from all cores and all depths.

1.4. Pyrotag sequencing

DNA was prepared for pyrotag sequencing using primers for the V6-V8 variable SSU rRNA region: 926f (5' AAACTYAAAKGAATTGRCGG 3') and 1392r (5' ACGGGCGGTGTGTRC 3') with adaptor and barcode, and PCR amplification using an iCycler® (Biorad) thermocycler under conditions: 95ºC for 3min; 25 cycles of 95ºC for 30s, 55ºC for 45s, 72ºC for 90s; and 72ºC for 10min. Amplicons were purified using the QIAquick® PCR purification kit (Qiagen). DNA concentrations and purity were measured on a NanoDrop® ND-2000 UV-Vis Spectrophotometer (NanoDrop Technologies, Wilmington, DE) and by running 1μL of the PCR product on a 0.8% agarose gel. Purified PCR products were sent to the McGill University Innovation Centre (Montréal, Québec, Canada) for pyrosequencing using a Roche GS-FLX Titanium Series sequencer. Pyrotag reads were filtered using the following quality control criteria: minimum length 200bp, no ambiguous base reads, no missing quality scores, mean quality score greater than 25, no more than 6 nucleotide length homopolymer runs and no mismatches in reverse primer. Raw pyrotag sequences were deposited in the sequence read archive of NCBI under project PRJNA239169.

References

Huber, T., Faulkner, G., Hugenholtz, P., (2004). Bellerophon: a program to detect chimeric sequences in multiple sequence alignments. *Bioinformatics* 20, 2317–9. doi:10.1093/bioinformatics/bth226

2. Supplementary Figures and Tables

2.1. Supplementary Tables

Supplemental Table 1: Putative sulfate-reducing bacteria found in the BCR pyrotag library.

OTU ID	Taxonomic assignment according to Silva 111 database		Read count	Nearest cultured relative	NCBI Accession number	Percent identity
OTU163	*Desulfobulbaceae*	*Desulfobulbus*	105	*Desulfobulbus elongatus* strain FP	NR_029305	99%
				Desulfobulbus propionicus strain DSM 2032	NR_074930	97%
OTU623	*Nitrospinaceae*	uncultured	14	none		
OTU181	*Desulfovibrionaceae*	*Desulfovibrio*	85	*Desulfovibrio idahonensis* strain CY1	NR_114908	99%
				Desulfovibrio arcticus strain B15	NR_115726	99%
				Desulfovibrio mexicanus strain Lup1	NR_028776	97%
OTU740	*Desulfovibrionaceae*	*Desulfovibrio*	12	*Desulfovibrio mexicanus* strain Lup1	NR_028776	99%
OTU673	*Desulfovibrionaceae*	*Desulfovibrio*	11	*Desulfovibrio mexicanus* strain Lup1	NR_028776	97%
OTU829	*Desulfovibrionaceae*	*Desulfovibrio*	9	*Desulfovibrio aminophilus* strain ALA-3	NR_024916	97%
OTU1597	*Desulfovibrionaceae*	*Desulfovibrio*	5	*Desulfovibrio gracilis* strain SRL6146	NR_044785	95%
OTU1650	*Desulfovibrionaceae*	*Desulfovibrio*	5	*Desulfovibrio idahonensis* strain CY1	NR_114908	92%
OTU1594	*Desulfovibrionaceae*	*Desulfovibrio*	4	*Desulfovibrio paquesii* strain SB1	NR_043145	98%
OTU600	*Peptococcaceae*	*Desulfosporosinus*	16	*Desulfosporosinus youngiae* strain JW/YJL-B18	NR_115694	97%
OTU807	*Peptococcaceae*	*Desulfosporosinus*	11	*Desulfosporosinus burensis* strain BSREI1	NR_109421	95%
OTU1525	*Peptococcaceae*	*Desulfosporosinus*	9	*Desulfosporosinus burensis* strain BSREI1	NR_109421	97%
OTU987	*Peptococcaceae*	*Desulfosporosinus*	7	*Desulfosporosinus orientis* strain DSM 765	NR_074131	95%

OTU1570	*Peptococcaceae*	*Desulfosporosinus*	4	*Desulfosporosinus burensis* strain BSREI1	NR_109421	95%
OTU954	*Peptococcaceae*	*Desulfotomaculum*	8	*Desulfotomaculum nigrificans* strain NCIMB 8395	NR_117718	100%

Supplemental Table 2: List of the highly correlated OTUs in the cluster associated with higher amounts of pyrolysable carbon, their taxonomic assignments and total read counts.

	**Taxonomic assignment**	**Read Count**
	**Domain;Phylum;Class;Order;Family;Genus**	
OTU147	Bacteria;Proteobacteria;Alphaproteobacteria;Sphingomonadales;Sphingomonadaceae;Novosphingobium	132
OTU47	Bacteria;Proteobacteria;Alphaproteobacteria;Rhizobiales;Hyphomicrobiaceae;Rhodoplanes	571
OTU1021	Bacteria;Proteobacteria;Alphaproteobacteria;Rhizobiales;Hyphomicrobiaceae;Hyphomicrobium	169
OTU57	Bacteria;Proteobacteria;Alphaproteobacteria;Rhizobiales;Xanthobacteraceae;Labrys	313
OTU6	Bacteria;Proteobacteria;Alphaproteobacteria;Rhizobiales;Rhodobiaceae;Rhodobium	3092
OTU10	Bacteria;Proteobacteria;Alphaproteobacteria;Rhizobiales;Hyphomicrobiaceae;Hyphomicrobium	3062
OTU977	Bacteria;Proteobacteria;Alphaproteobacteria;Rhizobiales;Rhodobiaceae;Rhodobium	260
OTU78	Bacteria;Proteobacteria;Alphaproteobacteria;Rhizobiales;Hyphomicrobiaceae;Pedomicrobium	340
OTU151	Bacteria;Proteobacteria;Alphaproteobacteria;Rhizobiales;Xanthobacteraceae;Azorhizobium	104
OTU74	Bacteria;Proteobacteria;Betaproteobacteria;Hot_Creek_32;Hot_Creek_32;Hot_Creek_32	170
OTU68	Bacteria;Proteobacteria;Betaproteobacteria;Burkholderiales;Comamonadaceae;Comamonadaceae	188
OTU484	Bacteria;Proteobacteria;Deltaproteobacteria;Sh765B-TzT-29;candidate_division_SBR1093;Sh765B-TzT-29	330
OTU21	Bacteria;Proteobacteria;Deltaproteobacteria;Sh765B-TzT-29;Sh765B-TzT-29;Sh765B-TzT-29	1217
OTU96	Bacteria;Proteobacteria;TA18;TA18;TA18;TA18	212
OTU13	Bacteria;Actinobacteria;Micrococcales;Dermatophilaceae;Dermatophilaceae;Dermatophilaceae	1895
OTU102	Bacteria;Actinobacteria;Thermoleophilia;Solirubrobacterales;Solirubrobacterales_480-2;	131
OTU37	Bacteria;Actinobacteria;Acidimicrobiia;Acidimicrobiales;TM214;TM214	618
OTU80	Bacteria;Actinobacteria;Acidimicrobiia;Acidimicrobiales;TM214;Acidimicrobidae_bacterium	188
OTU202	Bacteria;Actinobacteria;Acidimicrobiia;Acidimicrobiales;Acidimicrobiales;Acidimicrobiales	101
OTU138	Bacteria;Planctomycetes;Planctomycetacia;Planctomycetales;Planctomycetaceae;Planctomycetaceae	138
OTU72	Bacteria;Planctomycetes;Planctomycetacia;Planctomycetales;Planctomycetaceae;Planctomycetaceae	293
OTU24	Bacteria;Planctomycetes;Planctomycetacia;Planctomycetales;Planctomycetaceae;Pir4_lineage	929
OTU35	Bacteria;Bacteroidetes;Sphingobacteriia;Sphingobacteriales;PHOS-HE51;PHOS-HE51	608
OTU91	Bacteria;Bacteroidetes;Cytophagia;Cytophagales;Cytophagaceae;Flexibacter	164
OTU70	Bacteria;Bacteroidetes;Bacteroidia;Bacteroidales;Bacteroidaceae;Bacteroides	255
OTU16	Bacteria;Bacteroidetes;Bacteroidia;Bacteroidales;Prevotellaceae;Prevotella	1340
OTU7	Bacteria;Armatimonadetes;Armatimonadetes;Armatimonadetes;Armatimonadetes;Armatimonadetes	3207
OTU675	Bacteria;Fibrobacteres;Fibrobacteria;Fibrobacteria_KD2-123;Fibrobacteria_KD2-123;	93
OTU183	Bacteria;Chloroflexi;Anaerolineae;Anaerolineales;Anaerolineaceae;Longilinea	144

OTU35	Bacteria;Bacteroidetes;Sphingobacteriia;Sphingobacteriales;PHOS-HE51;PHOS-HE51	608
OTU55	Bacteria;Firmicutes;Clostridia;Clostridiales;Ruminococcaceae;Incertae_Sedis	498
OTU85	Bacteria;Firmicutes;Clostridia;Clostridiales;Ruminococcaceae;Ruminococcaceae	313
OTU17	Bacteria;Firmicutes;Clostridia;Clostridiales;Ruminococcaceae;Ruminococcaceae	1935
OTU175	Bacteria;Firmicutes;Clostridia;Clostridiales;Ruminococcaceae;Ruminococcaceae	1235
OTU30	Bacteria;Firmicutes;Clostridia;Clostridiales;Ruminococcaceae;Incertae_Sedis	1100
OTU991	Bacteria;Firmicutes;Clostridia;Clostridiales;Ruminococcaceae;Incertae_Sedis	94
OTU228	Bacteria;Firmicutes;Clostridia;Clostridiales;Ruminococcaceae;Ruminococcaceae	1151
OTU45	Bacteria;Firmicutes;Clostridia;Clostridiales;Ruminococcaceae;Incertae_Sedis	987
OTU42	Bacteria;Firmicutes;Clostridia;Clostridiales;Clostridiaceae;Clostridium	487
OTU1011	Bacteria;Firmicutes;Clostridia;Clostridiales;Ruminococcaceae;Incertae_Sedis	576
OTU93	Bacteria;Firmicutes;Clostridia;Clostridiales;Ruminococcaceae;Incertae_Sedis	312

2.2. Supplementary Figures

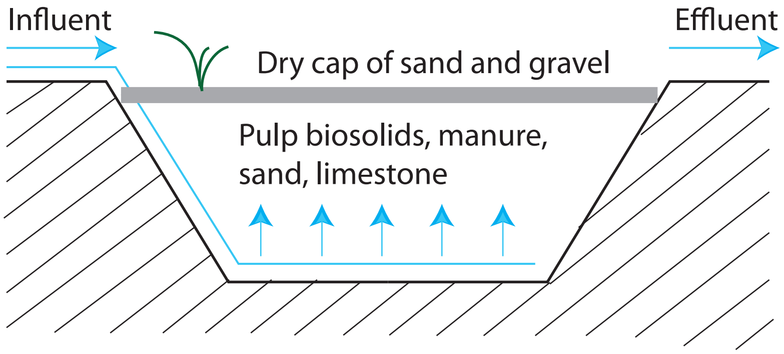

Supplemental Figure 1: Cartoon of a vertical cross-section through the BCR.

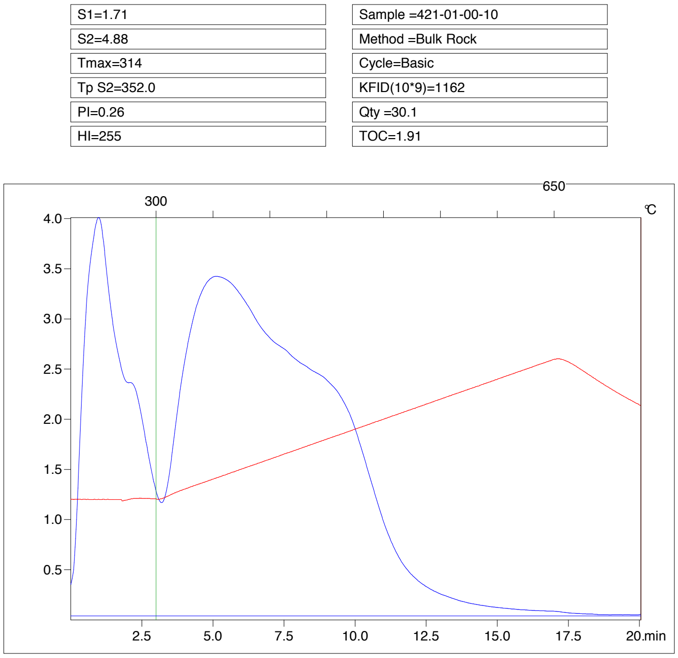

Supplemental Figure 2(a)

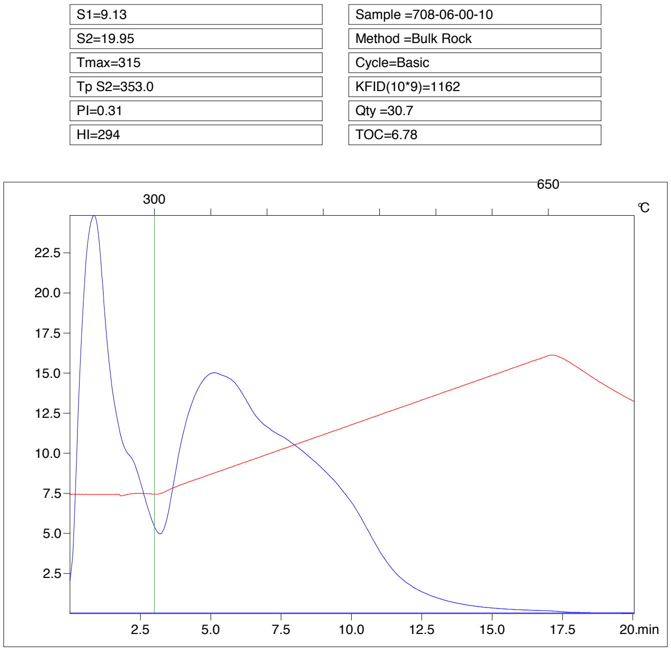

Supplemental Figure 2(b)

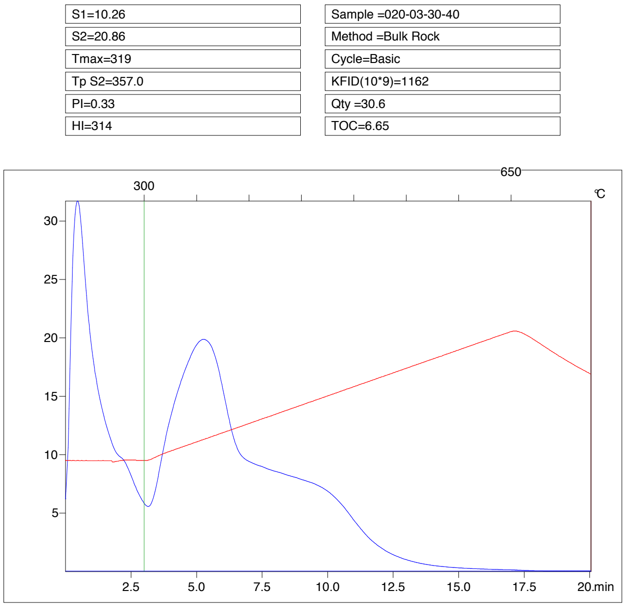

Supplemental Figure 2(c)

Supplemental Figure 2: Example Rock-Eval-6 curves for three values of pyrolysable carbon normalized with total organic carbon (PC_TOC): (a) 0.34, (b) 0.40 and (c) 0.45. These are the Flame Ionization Detector (FID) pyrograms showing the total concentrations of hydrocarbon released from the samples (mg hydrocarbon (HC)/g solid) versus time and ramping temperature (300 to 650°C at 25°C per minute). Y-axis is mg HC/g solid, x axis below is Time (minutes), and top is temperature

(°C).

Brief description of the procedure: The sample is heated to 300°C under an inert atmosphere during which volatile components are released and quantified (mg hydrocarbons (HC) per g solids) using flame ionization detection (FID). This constitutes the S1 fraction (small volatile compounds). This is represented by the area under the first peak of the plots in Figure 2. Then temperature is increased by 25°C/min to 650°C during which the compounds liberated by cracking are quantified, also using FID, as the S2 fraction (mg-HC/g-solids), which is the area under the second peak. The mg g^{-1} solids CO and CO_2 evolved during this period are recorded and comprise the S3 fraction deemed to represent oxygen-containing compounds. The total of the S1, S2 and S3 fractions is called pyrolysable carbon (PC wt%). A T_{peak} is recorded, which is the temperature at which the maximum peak of S2 material is liberated. Finally, the amount of remaining sample combusted under oxygen is called residual carbon (RC wt%). Total organic carbon (TOC wt%) is sum of RC and PC. A T_{peak} is recorded, which is the temperature at which the maximum peak of S2 material is liberated. MINC is the amount of mineral carbon present in the sample.

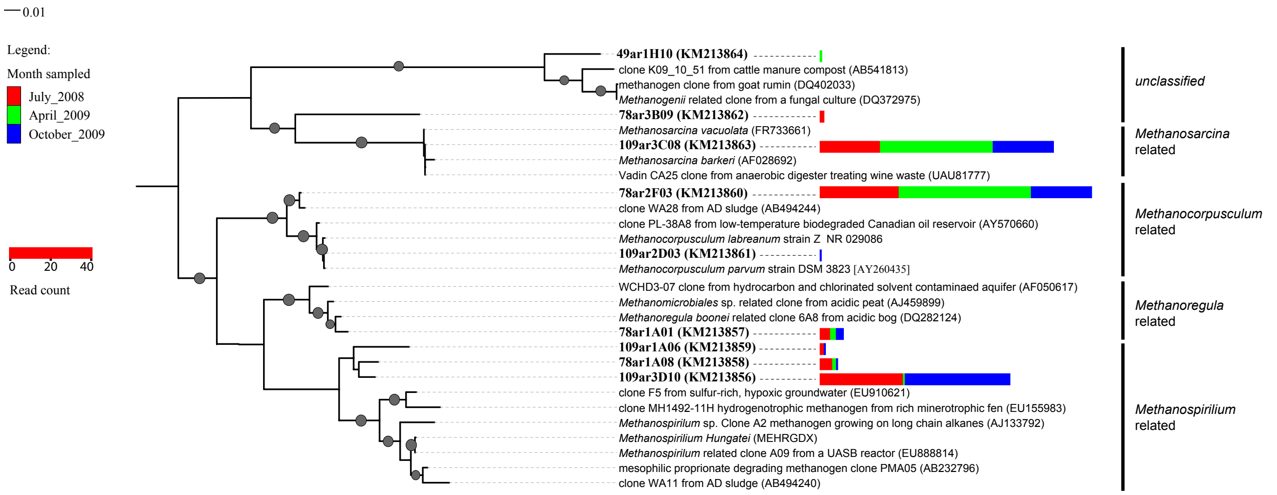

Supplemental Figure 3: Phylogenetic tree of the Archaea clone library 97% homology cut-off OTUs. Horizontal stacked colored bars depict the number of reads assigned to each OTU in samples removed during the three different sampling periods. Length of the colored bar is linearly proportional to the read count. Tree branches with greater than 75% bootstrap values are labeled with symbols. Final tree produced in the Interactive Tree of Life (iToL http://itol.embl.de/).

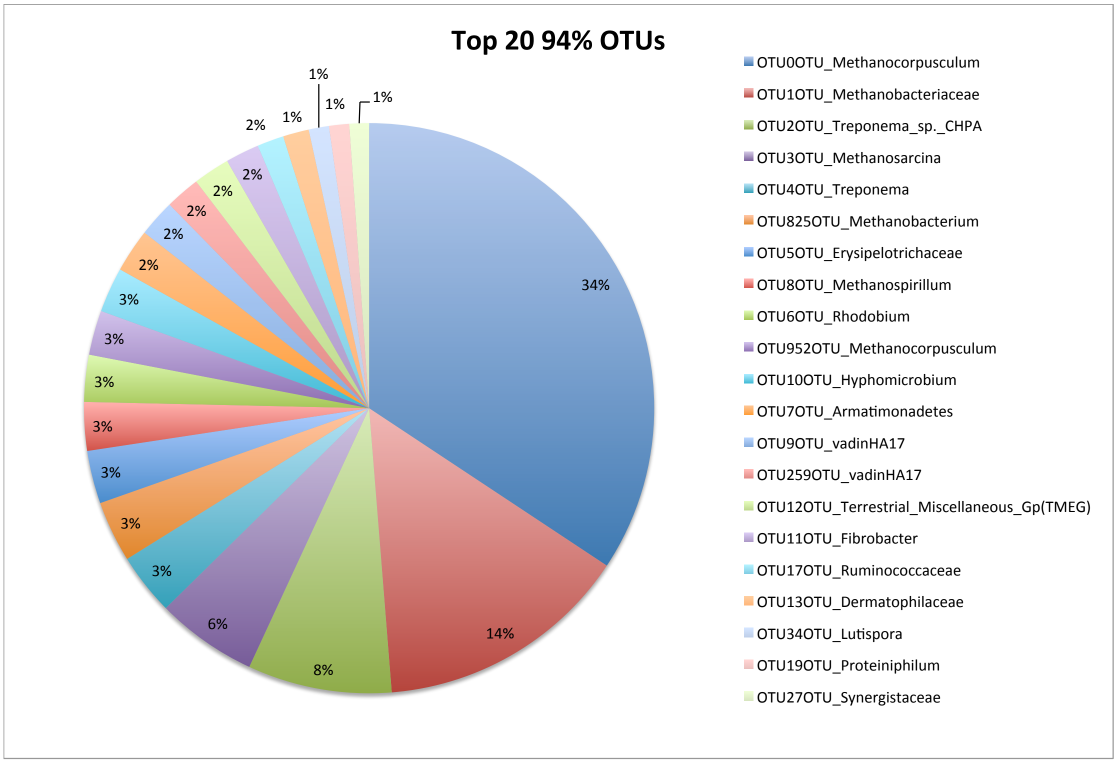

Supplemental Figure 4: Top 20 most highly represented 94% homology cut-off OTUs in the BCR according to the pyrotag reads. Colors in the legend from top to bottom represent colors on the pie chart in the clockwise direction starting at noon.

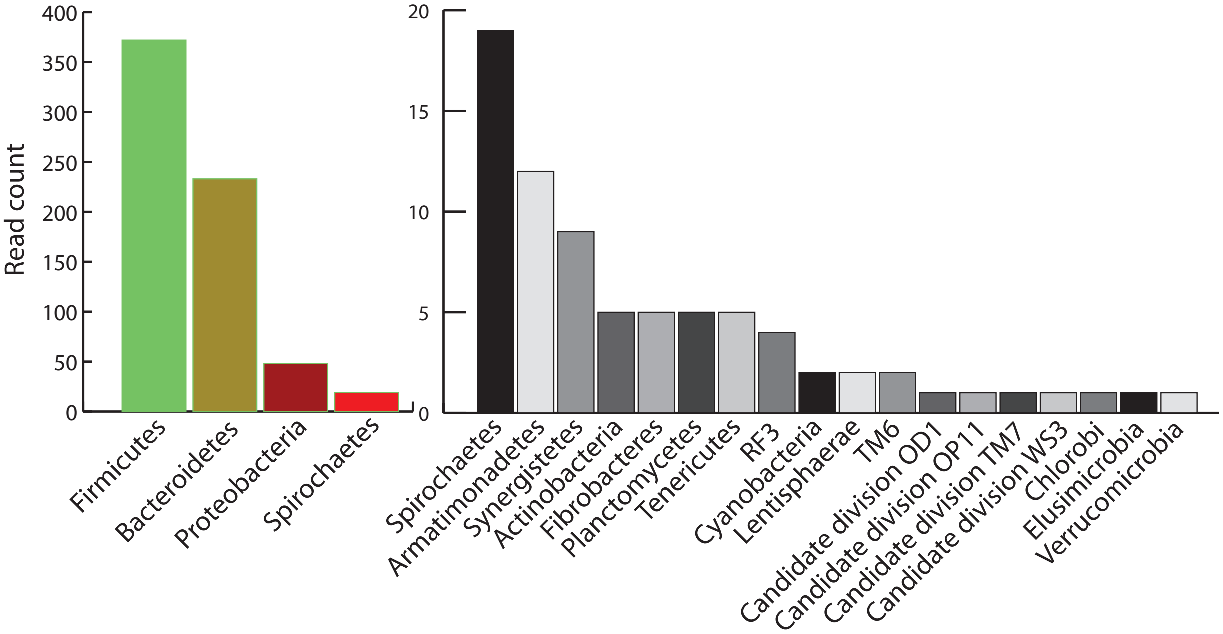

Supplemental Figure 5. Bacteria phyla represented in the BCR according to the clone library SSU rRNA sequences.

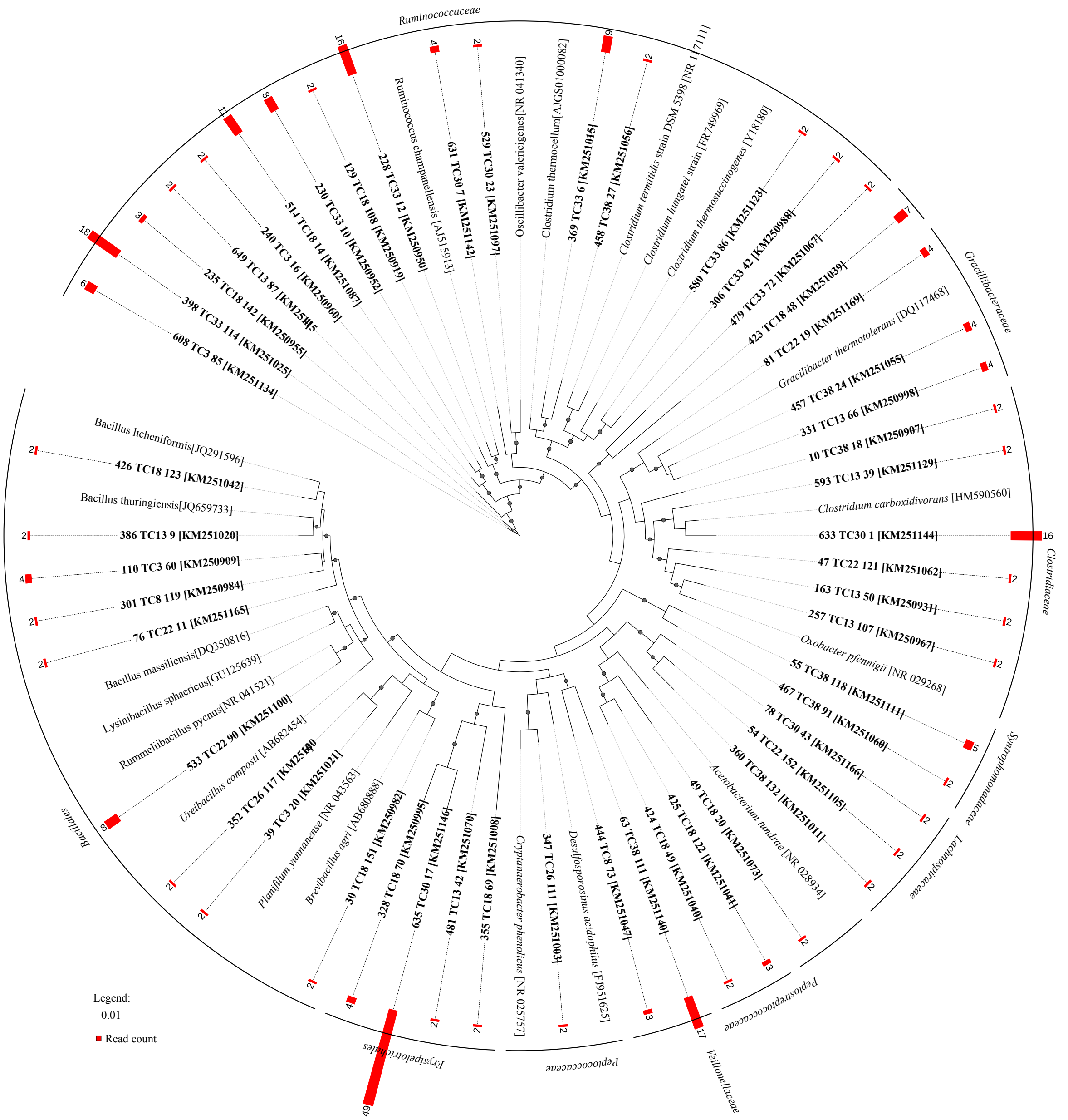

Supplemental Figure 6: Firmicutes clone library representative sequences and closely related cultured species. The lengths of the red bars represent their abundance in the clone library (number also given above the bar). Symbols on the branches indicate bootstrap values greater than 75%.

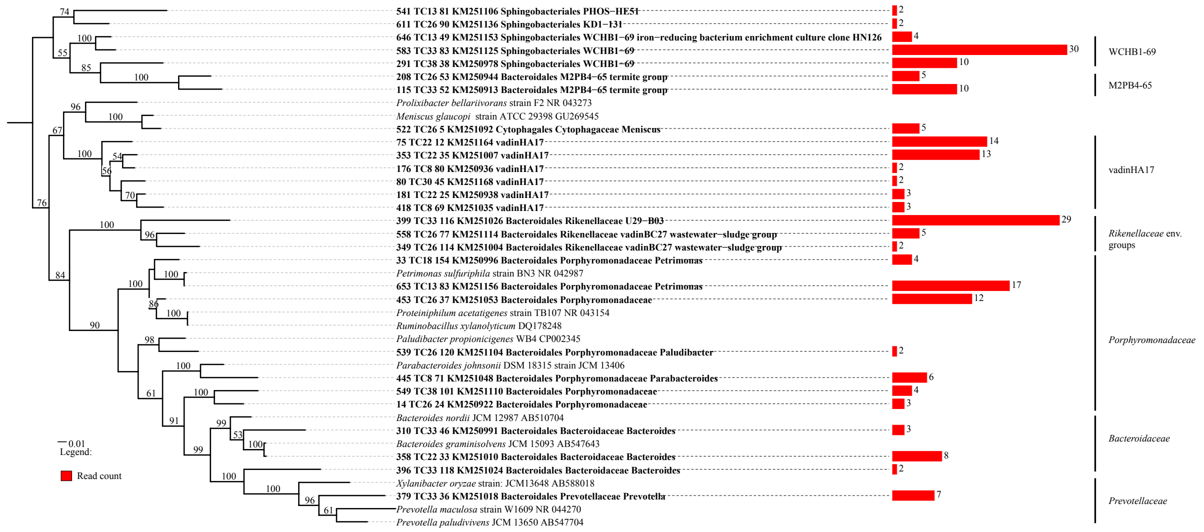

Supplemental Figure 7: Phylogenetic tree of clone library sequences classified in the Bacteroidetes phylum and closely related cultured species retrieved from the NCBI database. Class, order, family and genus level taxonomic classification according to the Silva 111 database given for each OTU. The number of reads included in each OTU is indicated by the length of the red bars on the right hand side.

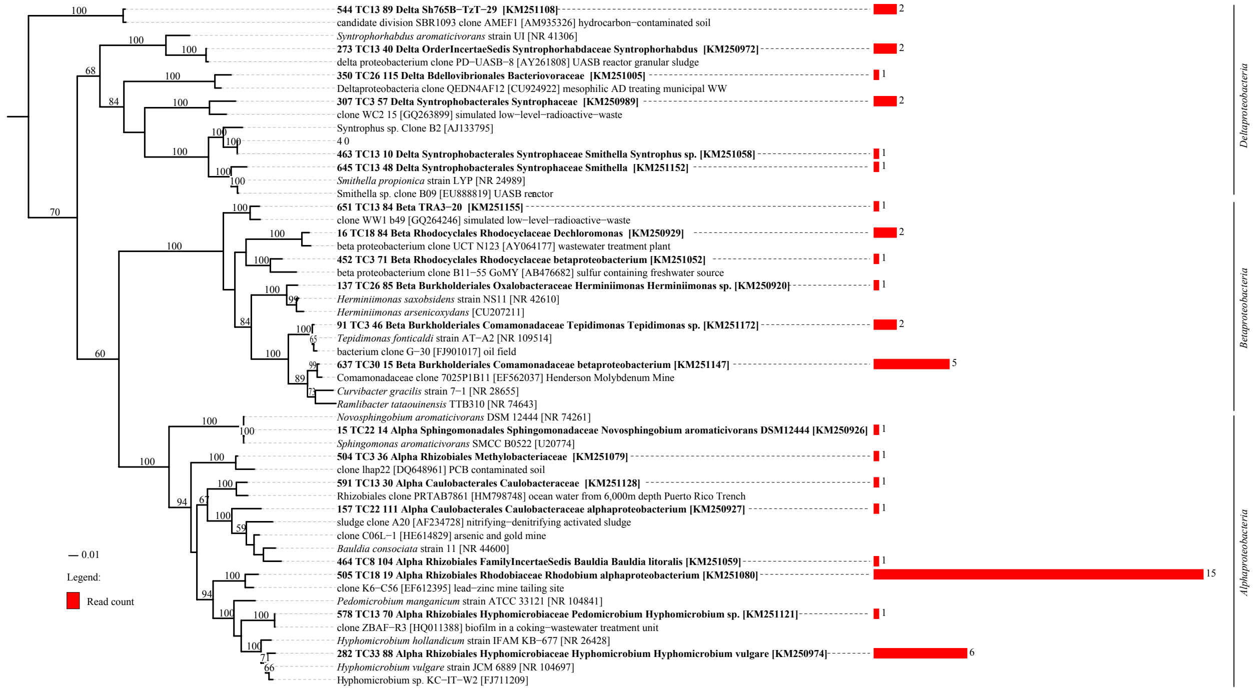

Supplemental Figure 8: Proteobacteria clone library representative sequences and closely related cultured species and environmental clones. Length of red bars represents read count. Symbols on branches indicate bootstrap values great than 75%.

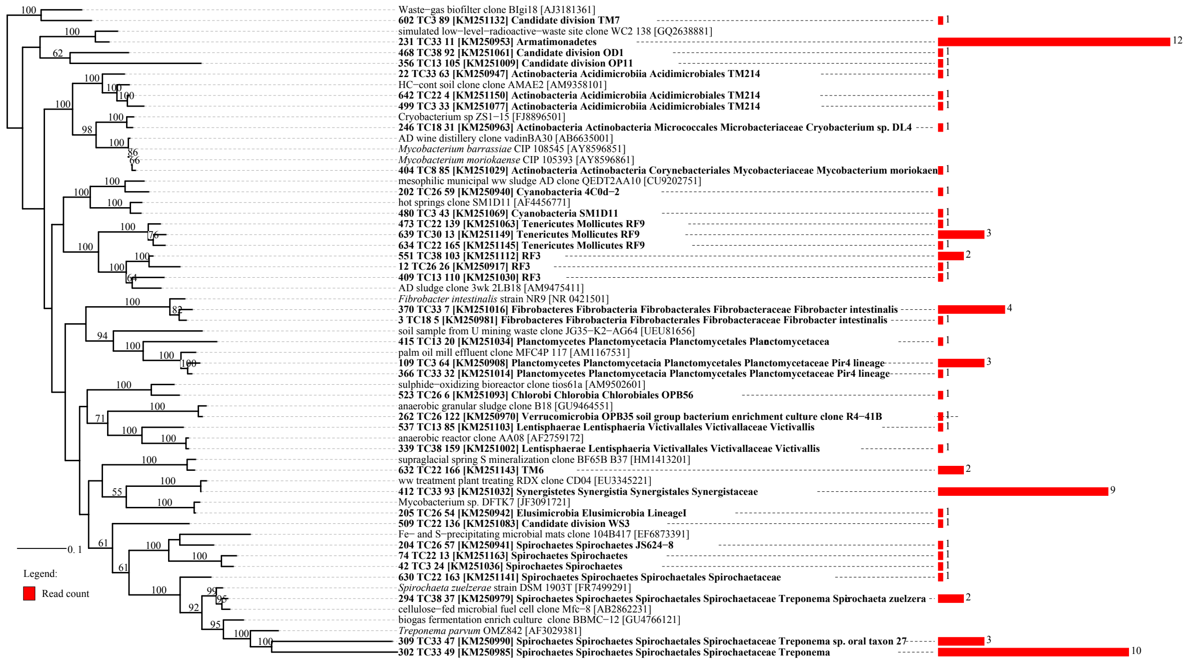

Supplemental Figure 9: Phylogenetic tree of clones classified in other phyla. Red bars represent the read counts in each 97% homology cut-off OTU.

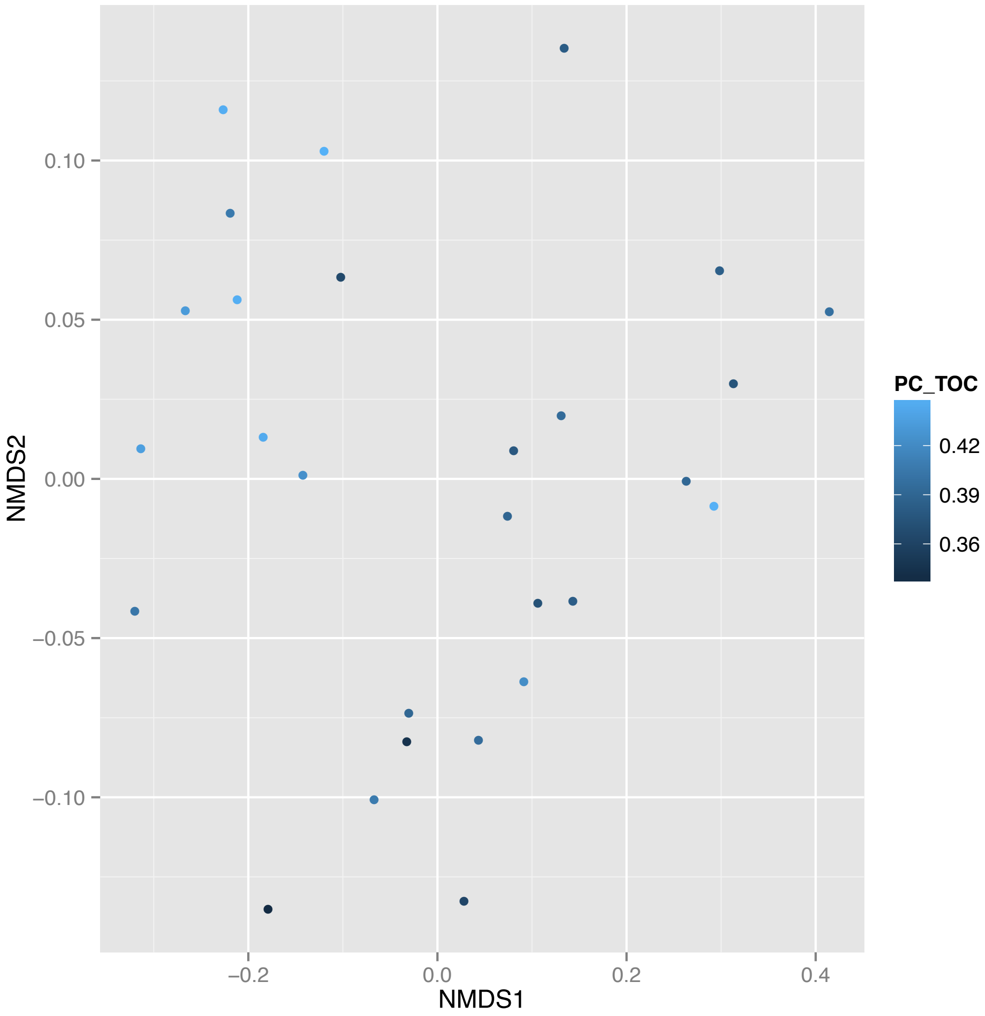

Supplemental Figure 10: Comparison of microbial community diversity between samples visualized on a non-metric dimensional scaling plot. Dots represent samples. Microbial community differences based on UNIFRAC analysis of phylogeny. Dots coloured according to the amount of pyrolysable carbon in the sample.

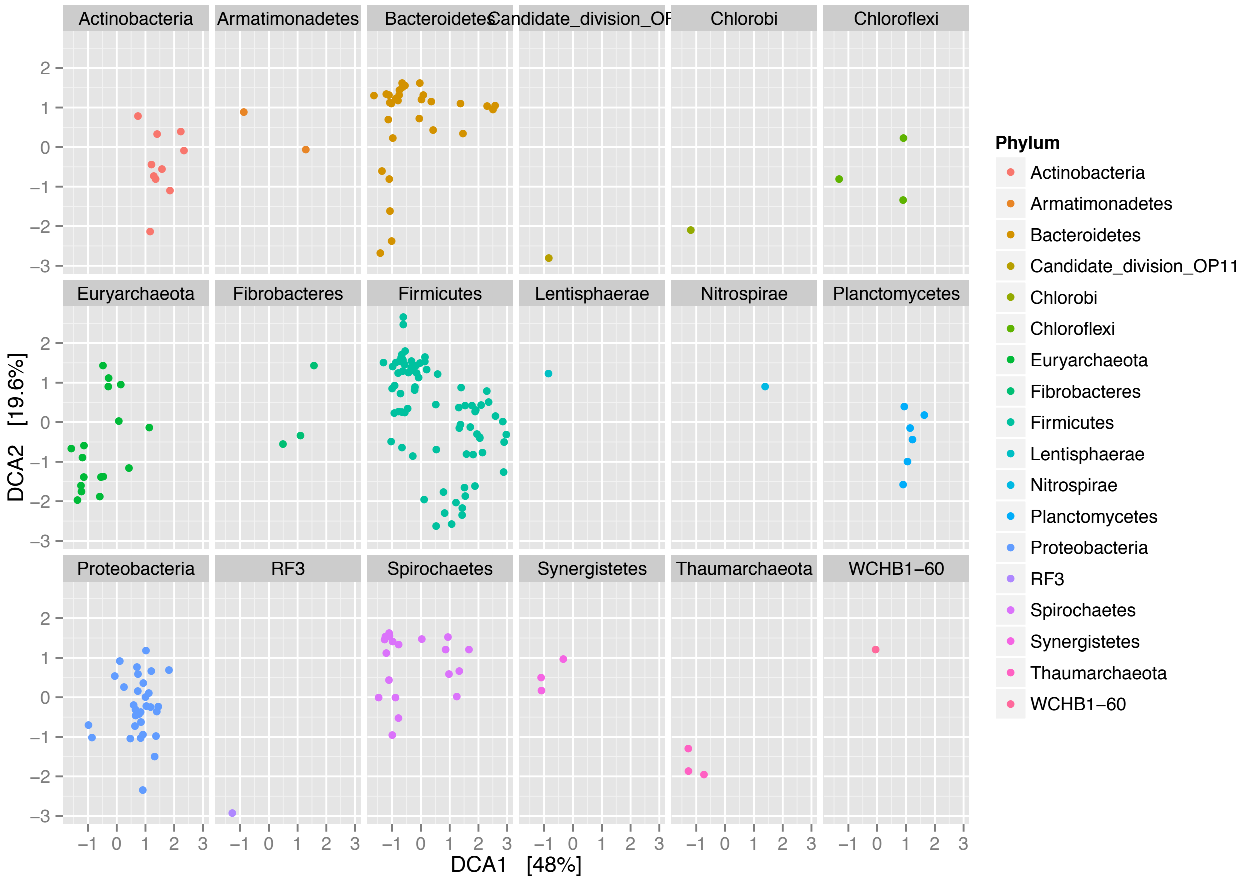

Supplemental Figure 11: Detrended correspondence plot of Bray-Curtis dissimilarity analysis of BCR core sample microbial community comparison showing OTUs split into facets according to Phylum.

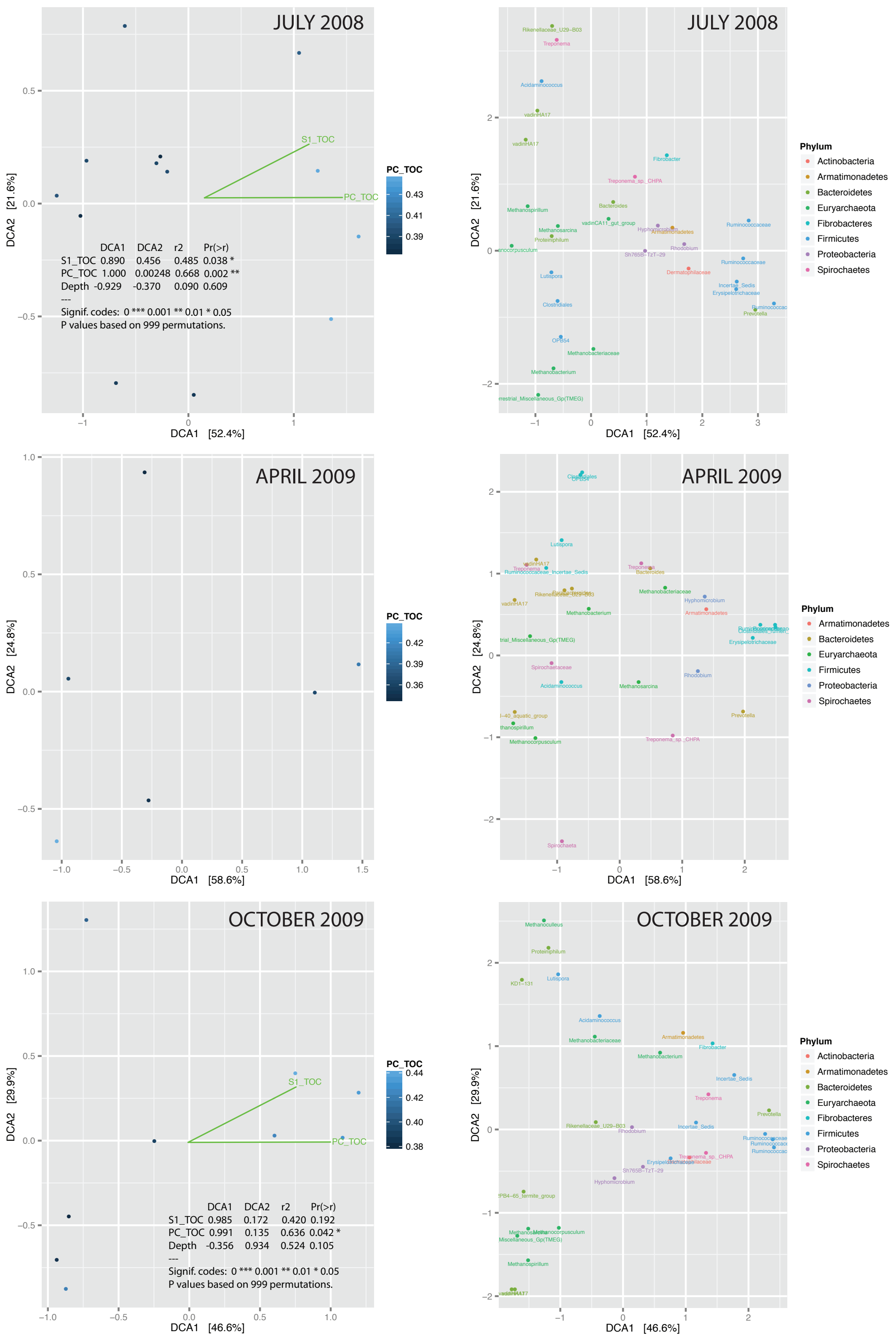
JULY 2008
S1_TOC
PC_TOC
PC_TOC
DCA1 DCA2 r2 Pr(>r)
S1_TOC 0.890 0.456 0.485 0.038 *
PC_TOC 1.000 0.00248 0.668 0.002 **
Depth -0.929 -0.370 0.090 0.609

Signif. codes: 0 *** 0.001 ** 0.01 * 0.05
P values based on 999 permutations.
DCA1 [52.4%]
DCA2 [21.6%]
JULY 2008
Phylum
Actinobacteria
Armatimonadetes
Bacteroidetes
Euryarchaeota
Fibrobacteres
Firmicutes
Proteobacteria
Spirochaetes
APRIL 2009
PC_TOC
DCA1 [58.6%]
DCA2 [24.8%]
APRIL 2009
Phylum
Armatimonadetes
Bacteroidetes
Euryarchaeota
Firmicutes
Proteobacteria
Spirochaetes
OCTOBER 2009
S1_TOC
PC_TOC
PC_TOC
DCA1 DCA2 r2 Pr(>r)
S1_TOC 0.985 0.172 0.420 0.192
PC_TOC 0.991 0.135 0.636 0.042 *
Depth -0.356 0.934 0.524 0.105

Signif. codes: 0 *** 0.001 ** 0.01 * 0.05
P values based on 999 permutations.
DCA1 [46.6%]
DCA2 [29.9%]
OCTOBER 2009
Phylum
Actinobacteria
Armatimonadetes
Bacteroidetes
Euryarchaeota
Fibrobacteres
Firmicutes
Proteobacteria
Spirochaetes

Supplemental Figure 12: Principal component detrended correspondence analysis (DCA) of the Bray-Curtis dissimilarity comparison of microbial community structure for each of the seasons (July 2008 (top panels), April 2009 (middle panels) and October 2009 (bottom panels)). Green lines on the left-hand-side panels depict the effect of environmental parameters S1 and PC on the microbial community composition. Statistical parameters output by the function envfit() in the R(vegan) package are included on the July and October panels since statistically significant fits were obtained of microbial community composition with PC and, to a lesser extent, S1. No statistically significant fit of microbial community structure and any of the environmental parameters was found for the samples collected in April. Right-hand-side panels indicate how the top 20 most prevalent 94% homology cut-off OTUs cluster, with methanogen-related OTUs towards the left together with samples containing less PC, and *Proteobacteria*- and *Firmicutes*-related OTUs towards the right together with samples with more PC (for the July and October samples).

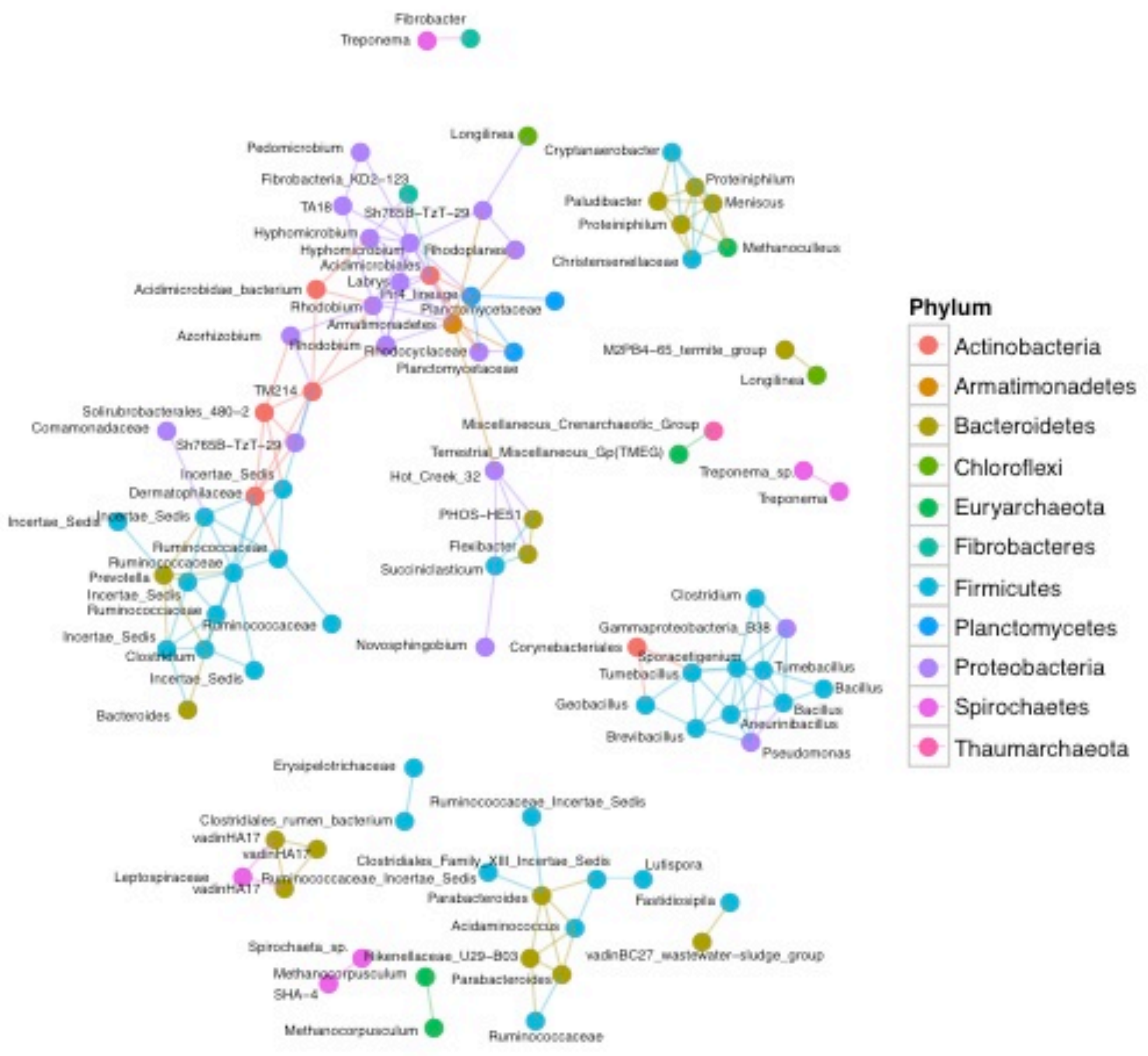

Supplemental Figure 13: Network diagram showing highly correlated 94% cut-off OTUs (edges shown for Pearson Correlation > 0.8 and p-value < 0.05). Each vertex represents an OTU and is labeled with its taxonomic assignment at the lowest meaningful taxonomic level. OTUs are coloured according to their Phylum.
